# Conventional and Novel Gγ Protein Families Constitute the Heterotrimeric G-Protein Signaling Network in Soybean

**DOI:** 10.1371/journal.pone.0023361

**Published:** 2011-08-10

**Authors:** Swarup Roy Choudhury, Naveen C. Bisht, Rheannon Thompson, Oleg Todorov, Sona Pandey

**Affiliations:** Donald Danforth Plant Science Center, St. Louis, Missouri, United States of America; University of Minnesota, United States of America

## Abstract

Heterotrimeric G-proteins comprised of Gα, Gβ and Gγ proteins are important signal transducers in all eukaryotes. The Gγ protein of the G-protein heterotrimer is crucial for its proper targeting at the plasma membrane and correct functioning. Gγ proteins are significantly smaller and more diverse than the Gα and Gβ proteins. In model plants *Arabidopsis* and rice that have a single Gα and Gβ protein, the presence of two canonical Gγ proteins provide some diversity to the possible heterotrimeric combinations. Our recent analysis of the latest version of the soybean genome has identified ten Gγ proteins which belong to three distinct families based on their C-termini. We amplified the full length cDNAs, analyzed their detailed expression profile by quantitative PCR, assessed their localization and performed yeast-based interaction analysis to evaluate interaction specificity with different Gβ proteins. Our results show that ten Gγ genes are retained in the soybean genome and have interesting expression profiles across different developmental stages. Six of the newly identified proteins belong to two plant-specific Gγ protein families. Yeast-based interaction analyses predict some degree of interaction specificity between different Gβ and Gγ proteins. This research thus identifies a highly diverse G-protein network from a plant species. Homologs of these novel proteins have been previously identified as QTLs for grain size and yield in rice.

## Introduction

Heterotrimeric G-proteins comprised of three dissimilar subunits α, β and γ are important signaling intermediates in all eukaryotes [1]–[3]. The Gα subunit, due to its ability to switch between the GDP-bound inactive form and GTP-bound active form, defines the status of signal transduction. Ligand binding to the GPCR causes a change in its conformation allowing an exchange of GTP for GDP on the Gα subunit [4]. The GTP-bound Gα dissociates from the Gβγ subunits and the released Gα•GTP and Gβγ dimer interact with a variety of effector proteins to transduce the signal. The intrinsic GTPase activity of Gα causes GTP hydrolysis, converting it back to its GDP-bound state. The Gα•GDP reassociates with the Gβγ dimer and the proteins return back to trimeric conformation [4], [5].

A wide range of fundamental signal transduction pathways are mediated via G-proteins in both plants and animals [4], [6]. In non-plant systems the multiplicity of each of the G-protein subunits, together with almost one thousand GPCRs, tissue-specific expression and signal-dependent heterotrimer formation, provides for the specificity of response [7], [8]. In plants the repertoire of G-protein components is relatively simple; the two most investigated species *Arabidopsis* and rice have only a single Gα, a single Gβ and two canonical Gγ proteins [9]. Given the presence of a single Gα and Gβ, the specificity in *Arabidopsis* and rice G-protein signaling is provided solely by the multiplicity of Gγ proteins.

We recently carried out an analysis of the soybean genome to assess if this paucity of G-protein components in plants is the norm and whether structural and functional diversity exists within the multiple copies of a gene present in highly duplicated genomes. Our analysis revealed a much more diverse plant G-protein family with the soybean genome encoding for four Gα, four Gβ and two Gγ proteins [10]. The number of Gα and Gβ proteins in the soybean genome corresponds well to the two recent genome duplication events [11] resulting in four copies of each gene. Interestingly both Gα and Gβ proteins exhibit some degree of interaction specificity between them. Moreover, based on the GTP-binding and GTPase activity, the four Gα proteins form two distinct subgroups. These data thus revealed that the G-protein signaling in plants is significantly more diverse and complex than what was predicted based on the studies in *Arabidopsis* and rice [10].

The presence of only two Gγ proteins in the soybean genome however did not correspond to what was expected based on the genome duplication events. Additionally two of the Gβ proteins GmGβ1 and GmGβ3 did not show any interaction with the GmGγ1 and GmGγ2 proteins, suggesting that additional Gγ proteins may exist. The small size and relatively low sequence conservation make the homology-based identification of Gγ proteins difficult; however, they do have certain conserved features to them. All known Gγ proteins contain a signature DPLL/I motif which together with few additional conserved amino acids in the middle coiled-coil region is required for interaction with the Gβ proteins. Most of the known Gγ proteins also contain a CAAX motif at C termini which is isoprenylated, resulting in the targeting of the proteins to the plasma membrane [12], [13].

In plants Gγ proteins have been reported from *Arabidopsis*, rice, pea and soybean. The *Arabidopsis* AGG1 and AGG2 proteins show 48% sequence identity and are involved in regulation of defense responses of plants [14]–[16]. These two proteins are the prototypical plant Gγ proteins. The rice RGG1 and soybean GmGγ1 and GmGγ2 proteins are highly homologous to the AGG1 protein and contain all the conserved features and motifs of Gγ proteins. The rice RGG2 protein is relatively diverse as this protein has an extra 57 amino acid extension at its N terminus (compared to RGG1) and does not contain the signature prenylation motif. The two reported pea Gγ proteins PGG1 and PGG2 are somewhat unusual as they do not contain the highly conserved DPLL/I motif even though a possible prenylation motif is present at its C termini [17]. The function of plant Gγ proteins has been evaluated only in *Arabidopsis* where the proteins participate in known Gα and/or Gβ mediated signaling pathways. Molecular-genetic analysis of knockout mutants in AGG1 and AGG2 reveals that the proteins are involved in regulating response to fungal pathogens [16], [18].

With the availability of a newer version of the soybean genome assembly (phytozome.net v7) and the use of a series of careful genome annotation programs, we queried the soybean genome to identify additional Gγ protein sequences. Our analysis identified two more canonical Gγ proteins that are present on the not yet annotated genome regions as well as six additional, novel Gγ proteins. The proteins display a great degree of diversity and can be grouped into three distinct families based on sequence features: the archetypal Gγ proteins, the prenylation-less Gγ proteins and the cysteine-rich Gγ proteins. This study describes the identification of these three families of Gγ proteins from soybean, details its expression profile in comparison to the expression profile of the *GmGβ* genes and evaluates its interaction with the specific GmGβ proteins. The presence of three different families of Gγ proteins in a single plant species supports a highly elaborate and diverse G-protein signaling network as well as provides clues to plant-specific G-protein signaling mechanisms, distinct from what is known based on mammalian systems.

## Results

### Identification of additional canonical and novel Gγ proteins from the soybean genome

Our previous analysis of the soybean genome had identified only two Gγ proteins [10]. We performed a careful search with the newer version of the soybean genome and identified eight additional Gγ proteins. The presence of ten Gγ proteins together with four Gα and four Gβ proteins thus corresponds to a total of one hundred and sixty possible heterotrimeric combinations.

Two of the newly identified Gγ proteins are highly homologous to the previously identified GmGγ1 and GmGγ2 and show high sequence homology between them (Table 1). We named these proteins GmGγ3 and GmGγ4, respectively. Both these proteins are present on the regions of the soybean chromosomes that have not yet been annotated. GmGγ3 is present on the chromosome 20 between the protein coding regions Glyma20g33300.1 and Glyma20g33320.1. We subsequently annotated the locus for GmGγ3 as Glyma20g33310.1. The open reading frame of this gene along with the positions of introns is reported in Figure S1. GmGγ4 is present on the chromosome 10 between the protein coding regions Glyma10g32210.1 and Glyma10g32220.1. We annotated the locus for GmGγ4 as Glyma10g32215.1. Figure S2 details the sequence of this newly annotated gene with its exons and intron.

**Table 1:** Amino acid sequence identity (%) of three different families of soybean Gγ proteins.

	GmGγ1	GmGγ2	GmGγ3	GmGγ4	GmGγ5	GmGγ6	GmGγ7	GmGγ8	GmGγ9	GmGγ10
**GmGγ1**	***	**92.7**	**50.9**	**50.9**	**29.4**	**29.4**	**29.4**	**20.2**	**19.3**	**19.3**
**GmGγ2**		***	**51.9**	**51.9**	**31.2**	**31.2**	**31.2**	**19.3**	**18.3**	**18.3**
**GmGγ3**			***	**99.1**	**32.1**	**32.1**	**32.1**	**23.6**	**24.5**	**18.9**
**GmGγ4**				***	**32.1**	**32.1**	**32.1**	**23.6**	**24.5**	**18.9**
**GmGγ5**					***	**99.2**	**93.7**	**20.6**	**19.8**	**19.1**
**GmGγ6**						***	**94.4**	**20.6**	**19.8**	**19.1**
**GmGγ7**							***	**21.4**	**19.0**	**20.6**
**GmGγ8**								***	**57.3**	**27.0**
**GmGγ9**									***	**26.4**
**GmGγ10**										***

**Figure 1:**
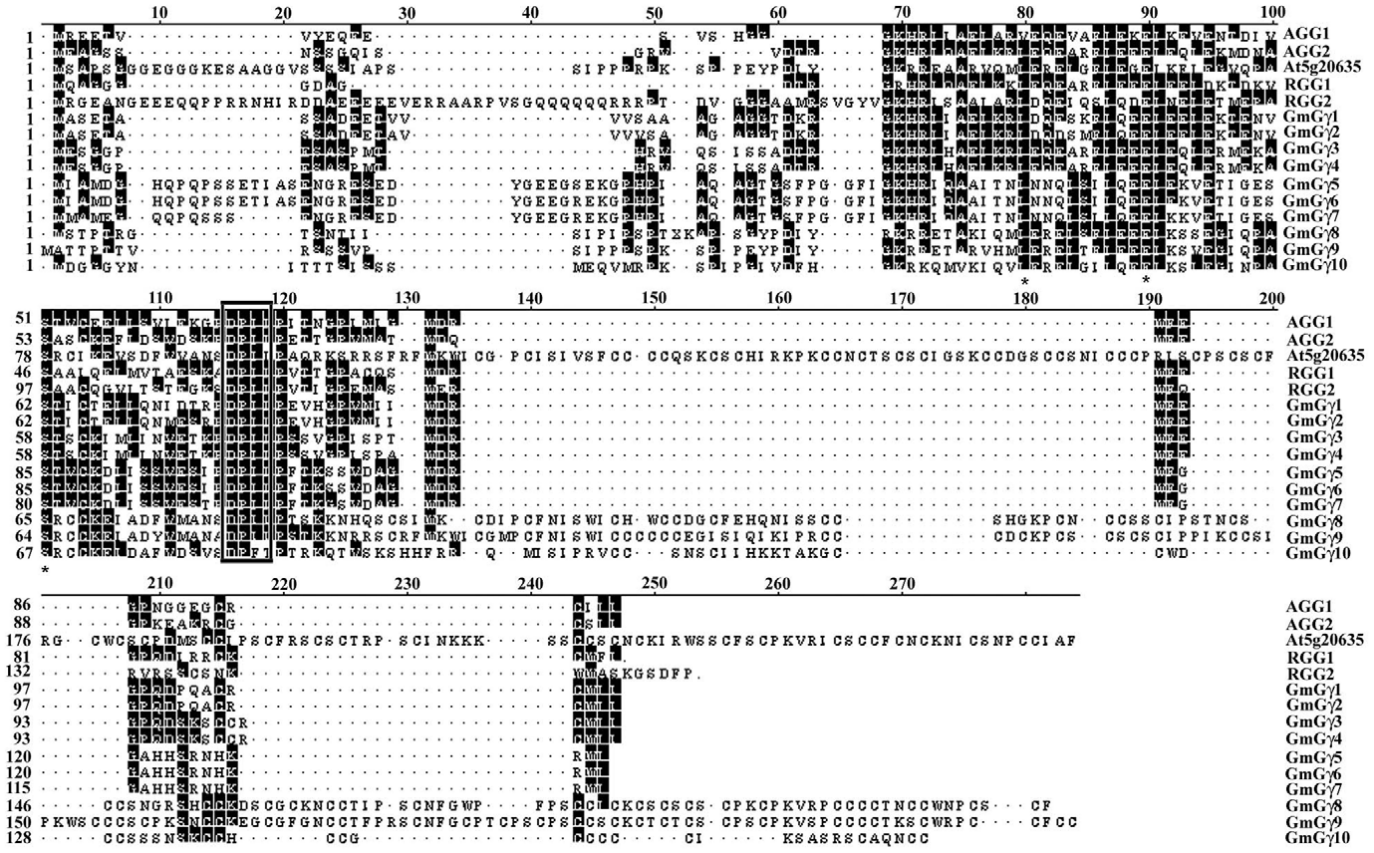
Amino acid sequence alignment of GmGγ proteins. The sequence alignment of *Arabidopsis* AGG1, AGG2 and At5g20635, rice RGG1 and RGG2, and the ten GmGγ was performed using Clustal W (www.clustal.org). Consensus sequences for interaction with Gβ proteins are marked with asterisks. Box represents the highly conserved DPLL/I motif in Gγ proteins. The amino acid positions are numbered in accordance with the GmGγ1.

These four GmGγ proteins Gγ1, 2, 3 and 4 have all the features of canonical Gγ proteins, namely the coiled-coil domain in the middle with the conserved DPLL motif at position 66-69 and conserved L30, E40 and S51 (amino acid numbers according to GmGγ1). These conserved features are important for the interaction of Gγ proteins with the Gβ proteins [19]. GmGγ3 and GmGγ4 also contain the CWIL motif at its C termini. This is the most common isoprenylation motif present in all plant Gγ proteins. We assigned these four prototypical Gγ proteins to group I. Figure 1 shows the protein sequence and conserved motifs of these Gγ proteins.

BLAST analysis [20] of the soybean genome using the coiled-coil region of the Gγ proteins identified three additional Gγ-like proteins on loci Glyma11g18050.1, Glyma14g17060.1 and Glyma17g29590.1. We named these proteins GmGγ5, GmGγ6 and GmGγ7, respectively. These three proteins share a high degree of sequence similarity with each other (Table 1). Based on its unique features, we assigned these three proteins to group II. Compared to the group I proteins, the group II proteins have an extra N terminal extension of 20-25 amino acids. The middle coiled-coil region of these proteins is highly similar to the group I proteins (Figure 1). Sequence features predicted to be involved in Gβ-Gγ interaction are conserved in group II Gγ proteins [19]. The most distinct feature of these group II proteins is the lack of C-terminal isoprenylation motif. The proteins end in a RWI sequence instead of a CWIL sequence. The group II proteins are thus somewhat similar to the rice RGG2 proteins as RGG2 also has an N terminal extension (albeit longer, 57 aa) and lacks the prenylation motif [21]. The group II Gγ proteins are also small in size: GmGγ5 and GmGγ6 are encoded by 131 amino acids and GmGγ7 is encoded by 126 amino acids, similar to canonical Gγ proteins [5]. Surprisingly a prenylation motif-less Gγ protein is not present in *Arabidopsis*.

The *GmGγ5* gene sequence is mis-annotated in the current version of the soybean genome. The predicted protein based on the genome annotation is much smaller and does not have the first exon as identified in our study. The genomic arrangement and experimental validation support the presented version of *GmGγ5* as the correct version. The correct sequence of the gene is detailed in Figure S3.

We identified three additional Gγ-like novel proteins on loci Glyma15g19630.1, Glyma17g05640.1 and Glyma07g04510.1. We named these proteins GmGγ8, GmGγ9 and GmGγ10, respectively and assigned them to group III based on its distinctive features. The group III proteins are significantly larger than conventional Gγ proteins as GmGγ8, GmGγ9 and GmGγ10 are encoded by 213, 228 and 159 amino acids, respectively. The N terminal of group III proteins is similar to group I proteins and the middle coiled-coil domain is highly conserved (Figure 1). GmGγ8 and GmGγ9 have all the sequence features required for Gβ interaction. GmGγ10 is the only protein identified in our analysis that does not have the conserved DPLL motif, instead this protein contains a similar DPFT motif (Figure 1). It is interesting to note that a highly conserved sequence in mammalian Gγ proteins N62 P63, F64 (numbers based on human Gγ1) is not conserved in the plant Gγ proteins. This region is required for the GPCR-dependent conformational change in Gγ [22].

In addition to large size, there are other features that are unique to group III Gγ proteins. The proteins are predicted to have a TNFR (tumor necrosis growth factor receptor) signature and a long cysteine rich C-terminal region which is not found in any other known Gγ proteins to date. The C terminus of these proteins is quite variable. Counting from the conserved DPLL (DPFT in case of GmGγ10) motif, the C terminal of GmGγ8, GmGγ9 and GmGγ10 consists of 130, 145 and 74 amino acids, respectively. This variable region of group III proteins is unusually high in cysteine content: GmGγ8 contains 30% Cys (39 out of 130 amino acids), GmGγ9 contains 33% Cys (49 out of 145 amino acids) and GmGγ10 contains 26% Cys (19 out of 74 amino acids).

The sequence of *GmGγ8* and *GmGγ10* is mis-annotated in the current version of the soybean genome assembly. The predicted genes could never be amplified in our analysis. We manually annotated these genes and amplified the full length product. Based on the experimentally obtained cDNAs, we marked the correct exon-intron boundaries of *GmGγ8* (Figure S4) and *GmGγ10* (Figure S5).

We also identified a homolog of GmGγ9 in the *Arabidopsis* genome at locus At5g20635. This gene has been recently described as a Gγ protein in *Arabidopsis* [23]. Homologs of group III proteins are present in all plant species. The proteins also show some homology to a keratin associated protein present in mammals. Interestingly the homologs of group III proteins in rice which are named *DEP1* and *GS3* have been recently identified as major QTLs for grain size and yield determination [24], [25].

### Genome organization and phylogenetic relationship analysis of soybean Gγ proteins

**Figure 2:**
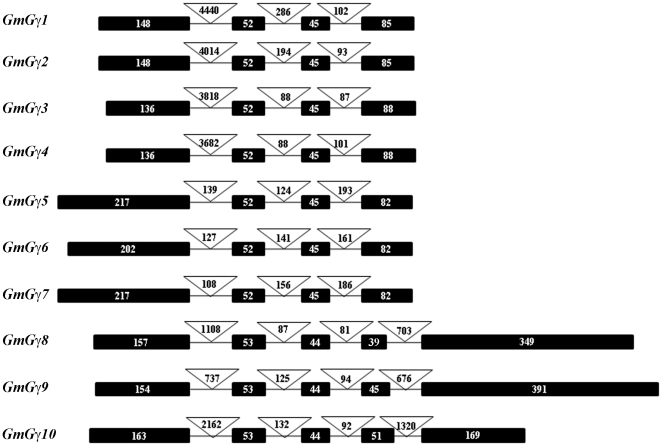
Exon-intron organization of the genomic regions coding for GmGγ proteins. Boxes represent exons and lines represent introns. The introns are not drawn to scale. Numbers denote the length of respective exons and introns.

The availability of multiple Gγ protein sequences in soybean with seemingly variable sequence features raised the question whether these protein families originated from the duplication of a single gene. We analyzed the chromosomal location of all ten *GmGγ* genes and the organization of exon and introns (Figure 2). Group I and group II *GmGγ* genes have four exons each, whereas five exons are present in the group III genes. The length of the second and third exons is highly conserved between all ten *GmGγ* genes. The second exon is 52 bp in group I and II and 53 bp in group III. Similarly, the third exon is 45 bp long in group I and II and 44 bp long in group III *GmGγ* genes. These two exons code for the highly conserved, middle coiled-coil domain of GmGγ proteins. This extreme conservation of the exon organization suggests that the proteins originated from this core sequence and acquired variable N- and C-terminal sequences. The group I and group II *GmGγ* genes also show a highly conserved fourth exon. It is also interesting to note that despite similarity in the size of cDNAs and proteins of the group I and group II GmGγs, the group I genes are encoded by large genomic regions (∼4–5 kb) and have a very long first intron ranging from 3.5 to 4.5 kb. This feature is not present in the group II *GmGγ* genes. The group III *GmGγ* genes show relatively less conserved genome organization even within the group. The last two exons of this group encode for the cysteine rich region of the proteins and display variability in size (Figure 2).

Phylogenetic analysis of all soybean Gγ proteins, along with the *Arabidopsis* and rice sequences, groups them in three expected subgroups with the two *Arabidopsis* proteins, the rice RGG1 and the GmGγ1-4 in one subgroup, the GmGγ group II proteins and rice RGG2 in another subgroup and the GmGγ group III proteins, the *Arabidopsis* At5g20635 and rice DEP1 and GS3 in the third subgroup (Figure S6). Analysis of chromosome location of *GmGγ* genes suggests that the four group I genes have resulted from two genome duplication events with the first event resulting in two related genes which duplicated again to result in highly homologous *GmGγ1* and *GmGγ2* forming one pair and *GmGγ3* and *GmGγ4* forming another. Similarly within group II, the genes *GmGγ5* and *GmGγ6* form a duplicated gene pair; and within group III, the genes *GmGγ8* and *GmGγ9* form a duplicated gene pair. We did not identify duplicated gene pairs corresponding to *GmGγ7* and *GmGγ10.* These might have been lost during evolution.

### Tissue-dependent expression analysis of soybean *Gγ* genes

In mammalian systems where multiple isoforms of Gγ proteins are present, a high degree of tissue specificity is observed for expression. Similarly the *Arabidopsis* Gγ proteins also show distinct expression patterns [16]. We assessed the expression profile of the ten *GmGγ* genes by real-time quantitative PCR to evaluate whether all ten *GmGγ* genes are expressed and the comparative expression levels. We quantified the absolute expression of each gene by a 100 fold serial dilution of cloned plasmid DNA and ascertained the specificity and efficiency of the individual primer pairs (Table S2, Figure S7). A linear correlation coefficient (*R^2^*) of 0.98-0.99 was observed over a 100,000 fold dilution. Interestingly unlike *Gα* and *Gβ* genes that are expressed at relatively similar levels in different tissue types [10], a range of variable expression levels were observed for the *GmGγ* genes.

**Figure 3:**
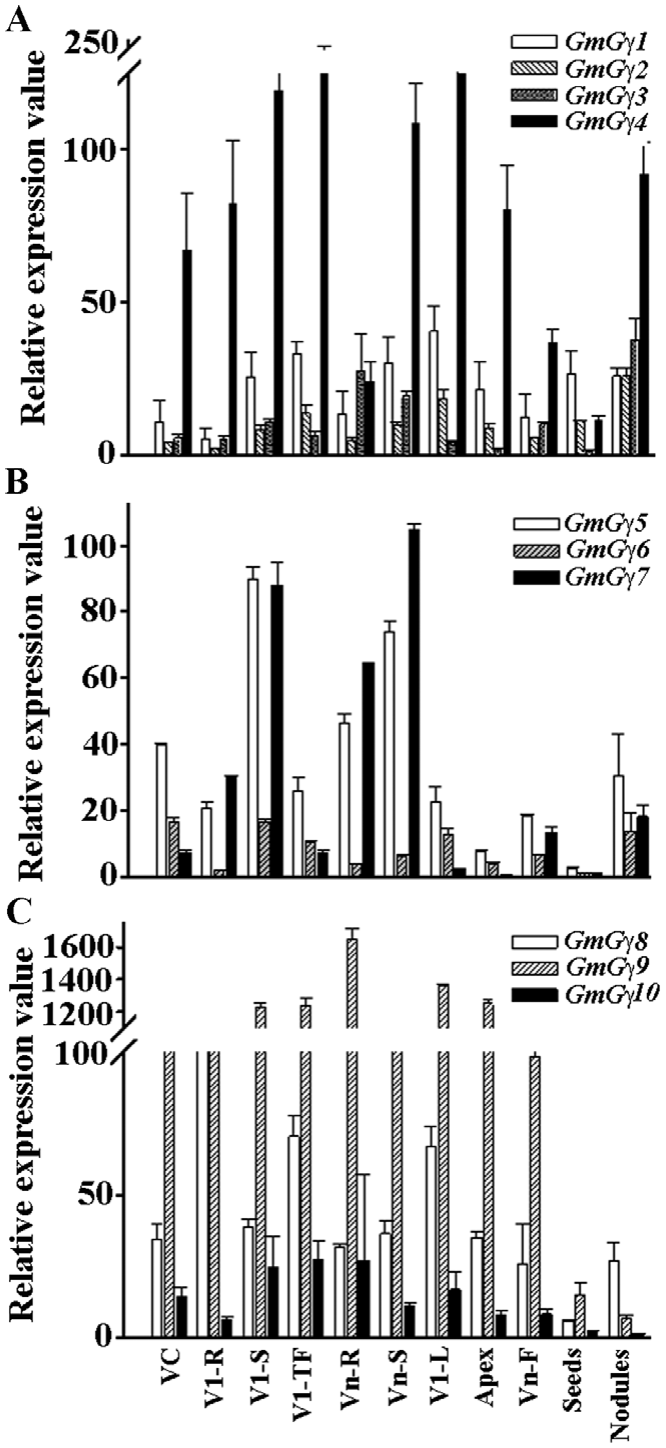
Expression of soybean *GmGγ* genes in different tissue types. The stages are defined as VC: cotyledon; V1-R: primary root at stage V1 (appearance of the first set of unfolded trifoliate leaves); V1-S: primary stem at stage V1; V1-TF: first trifoliate leaf; Vn-R: mature root; Vn-S: mature stem; Vn-L: mature leaves; Apex: inflorescence apex; Vn-F: flower; seeds: developing seeds at stage S4. qRT-PCR amplifications were performed thrice independently for each target, and the data were averaged. The expression values across different tissue types were normalized against soybean *Actin* gene expression [59], which was set at 100. Error bars represent the standard error of the mean. (A) Relative expression of the group I *GmGγ* genes. (B) Relative expression of the group II *GmGγ* genes. (C) Relative expression of the group III *GmGγ* genes.

We analyzed the tissue specific expression of the three families of *GmGγ* genes in vegetative tissues and reproductive tissues. Additionally given the possible role of G-protein dependent signaling during nodulation [26]–[30], we also analyzed the expression of *GmGγ* genes in this legume-specific tissue. Of the group I genes, *GmGγ4* exhibits overall high expression compared with the other members of this group (Figure 3A). In general, all four genes are expressed in all tissue types tested; however, the expression of *GmGγ4* is comparatively lower in roots than in aerial tissues whereas *GmGγ3* is most highly expressed in nodules and at a very low level in developing seeds (S4). The expression data of the genes *GmGγ1* and *GmGγ2* are presented for comparing relative expression level to the *GmGγ3* and *GmGγ4* genes.

Within the group II genes *GmGγ6* is expressed at a relatively low level compared to the other two members of this group (Figure 3B). *GmGγ7* in general has lower expression in leaves at all stages of development. All three genes are expressed at a moderate level in nodules. Additionally the group II genes are expressed at a relatively lower level in reproductive tissues *i.e.* the inflorescence apex, flowers and at an almost non-detectable level in seeds compared to the vegetative tissue (Figure 3B).

The group III genes show maximum variability in tissue specific expression. In this group *GmGγ9* is expressed at an extremely high level when comparing within this group or with any of the other *GmGγ* genes; whereas *GmGγ10* is expressed at a very low level. Noticeably this group of genes shows poor expression in nodules and seeds (Figure 3C).

### Expression of *GmGγ* genes during seed development and germination

Our previous gene expression analysis with the soybean Gα and Gβ genes showed interesting expression patterns during seed development and germination [10]. We analyzed the expression pattern of the newly discovered *GmGγ* genes during seed development and germination and compared it to the expression of *GmGβ* genes.

**Figure 4:**
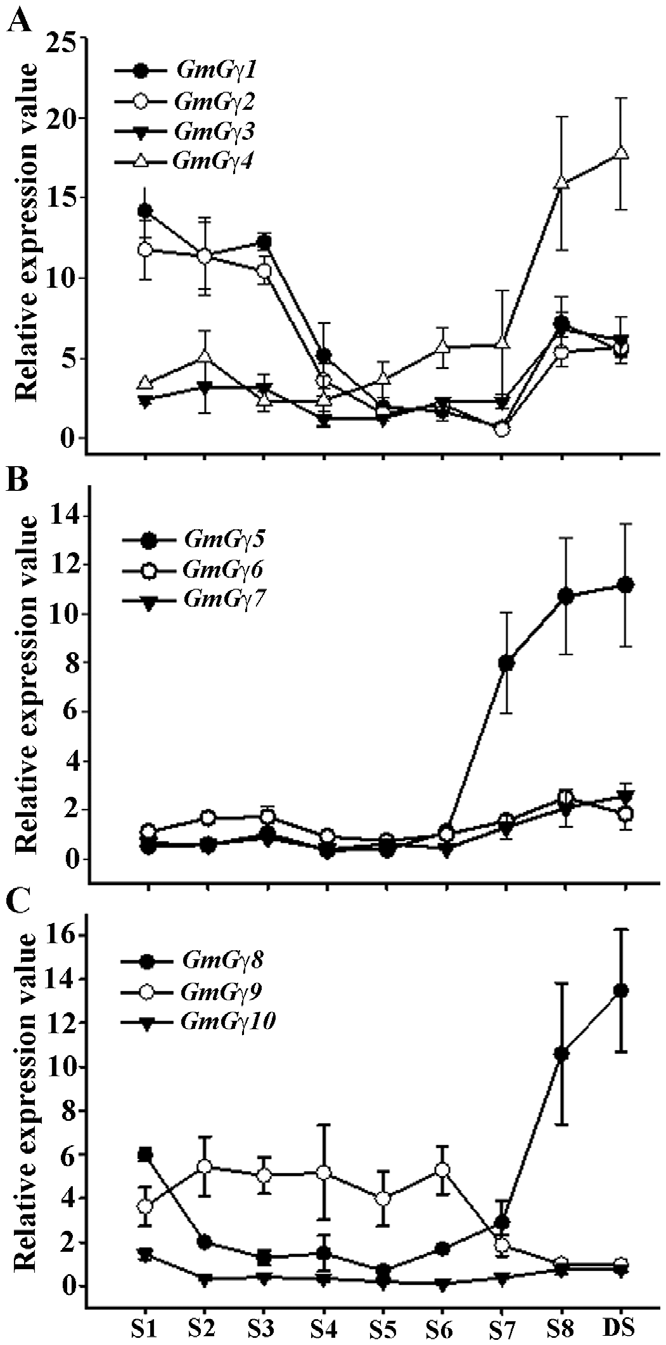
Expression of *GmGγ* genes during different stages of seed development. The seed development stages (S1-S8) are as described in [10]. qRT-PCR amplifications were performed thrice independently for each target, and the data were averaged. The expression values across different seed stages were normalized against soybean *Actin* gene expression. Dry seeds (DS) were also used for the analysis. Error bars represent the standard error of the mean. (A) Relative expression of the group I *GmGγ* genes during different stages of seed development. (B) Relative expression of the group II *GmGγ* genes. (C) Relative expression of the group III *GmGγ* genes.

Specific patterns of gene expression were observed for different *GmGγ* genes. For example *GmGγ3, GmGγ6* and *GmGγ7* do not show any change in expression during seed development (Figure 4) similar to *GmGβ1* and *GmGβ4* [10]; whereas *GmGγ1, GmGγ2* and *GmGγ9* show moderate down-regulation during seed maturation (Figure 4). Conversely, *GmGγ4, GmGγ5* and *GmGγ8* exhibit significant up-regulation of expression during seed maturation (Figure 4). These genes thus show expression profiles similar to *GmGβ2* and *GmGβ3*. It is also noticeable that the expression of these three Gγ genes, *GmGγ4, GmGγ5* and *GmGγ8,* is maintained at a high level in dry seeds whereas the expression of *GmGβ2* and *GmGβ3* genes returns back to basal levels in dry seeds [10].

**Figure 5:**
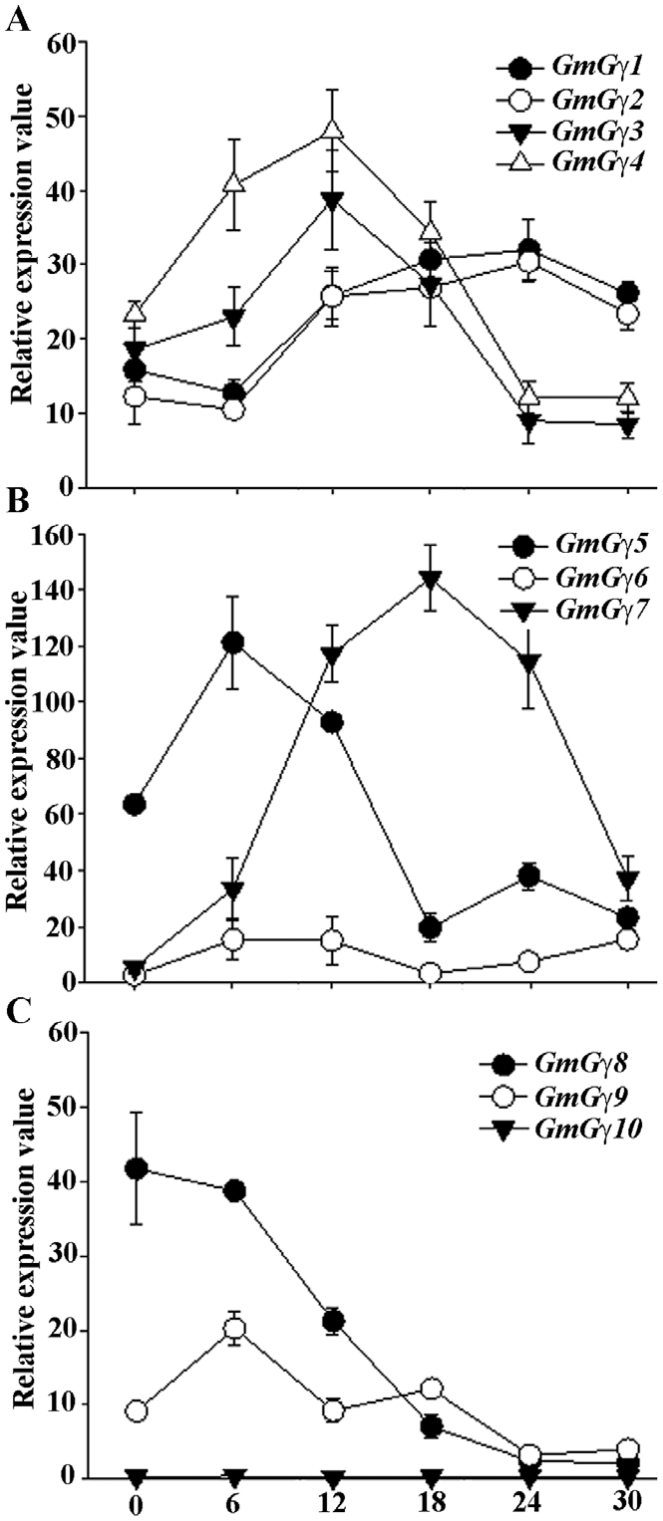
Expression of *GmGγ* genes during seed germination. Seed germination was followed starting from dry seeds (0 h) up to 30 h when an obvious radical had protruded. Seed samples were collected at every 6 h following imbibition. qRT-PCR amplification experiments were performed thrice independently for each target, and the data were averaged. The expression values across different seed germination stages were normalized against soybean *Actin* gene expression. Error bars represent the standard error of the mean. (A) Relative expression of the group I *GmGγ* genes during different stages of seed development. (B) Relative expression of the group II *GmGγ* genes. (C) Relative expression of the group III *GmGγ* genes.

G-proteins are involved in the regulation of seed germination in *Arabidopsis* [31], [32] and the soybean *GmGα* and *GmGβ* genes show distinct patterns of expression during different stages of seed germination [10]. *GmGγ3* and *GmGγ4* follow the similar pattern as *GmGβ3* and *GmGβ4* with higher expression starting 6 h after imbibition, maximizing at 12 h followed by a decrease in expression (Figure 5). The expression of *GmGγ5* and *GmGγ9* is similar to the expression of *GmGβ1* and *GmGβ2* with expression peaking at 6 h after imbibition followed by a decrease in expression (Figure 5). The genes *GmGγ6* and *GmGγ10* are expressed at a significantly low level in dry seeds and do not show any change in expression during germination (Figure 5). *GmGγ7* is also expressed at a very low level in dry seeds (Figure 4C); however, the expression of this gene is significantly up-regulated during germination. The genes show higher expression starting 6 h post-imbibition and maximum expression is observed at 24 h post imbibition followed by a gradual decrease. In contrast *GmGγ8* which is expressed at a high level in dry seeds shows a significant down-regulation in its expression following 6 h post imbibition.

We also tested the expression of different *GmGγ* genes in response to various stresses. No significant differences were observed in the expression of any of the genes under the conditions where a stress-marker gene *GmRab18* was expressed at significantly higher level (data not shown). These data are similar to what we earlier observed for the *GmGα* and *GmGβ* genes [10].

### Localization of soybean Gγ proteins

**Figure 6:**
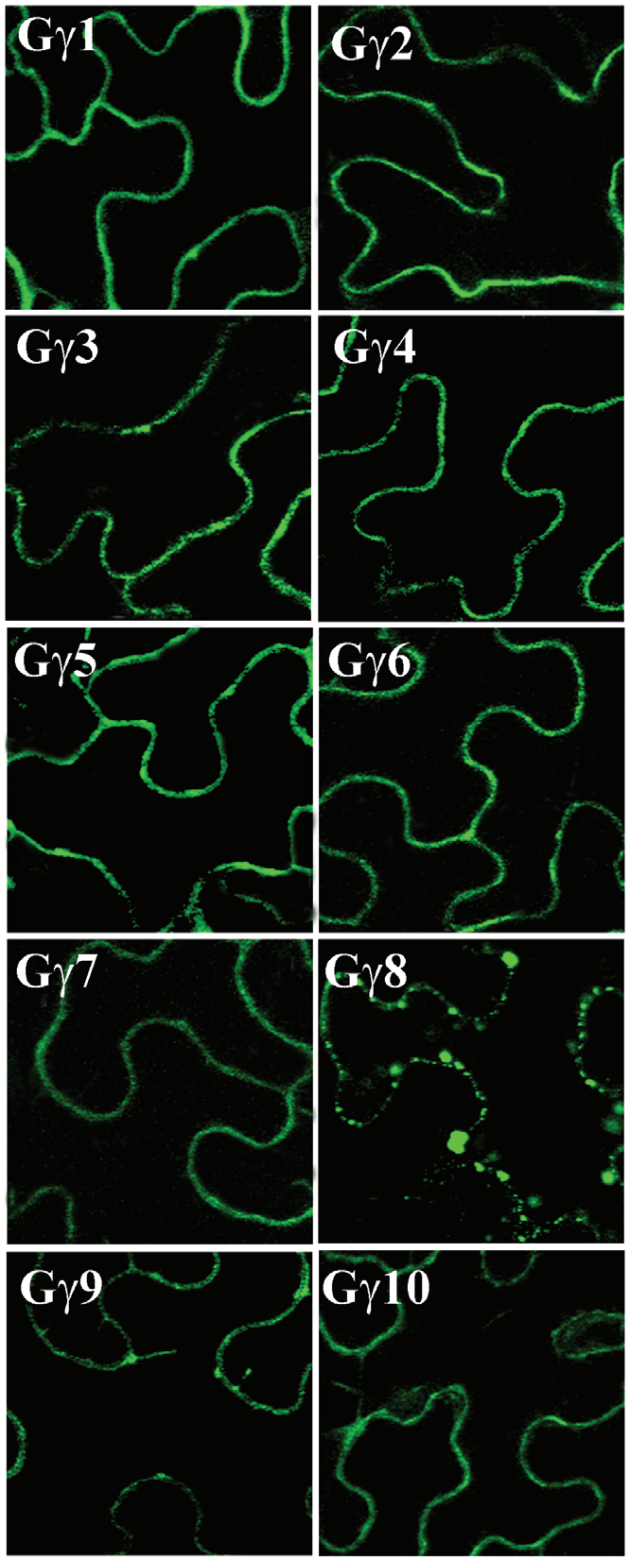
Localization of GmGγ proteins. Localization of *YFP:GmGγ* 1-10 genes in transiently transformed tobacco leaves using confocal microscopy. At least three independent transformations were performed. The figure shows representative picture from each transformation.

Canonical Gγ proteins are localized to the plasma membrane via the isoprenylation of C terminal sequence [33], [34]. The presence of three Gγ protein groups with distinctly variable C terminal sequences allowed us to assess whether the three groups of proteins exhibit any differences in localization. We transiently transformed tobacco leaves via agrobacterium-mediated transformation with YFP (yellow fluorescent protein) fused with respective *GmGγ* genes at N termini. The transformed leaves were visualized with confocal microscopy. The group I fusion proteins YFP-GmGγ1, YFP-GmGγ2, YFP-GmGγ3 and YFP-GmGγ4 showed fluorescence localized to the periphery of the cells as expected based on the presence of a canonical isoprenylation motif at the C terminal of these proteins (Figure 6). Similarly the group II fusion proteins YFP-GmGγ5, YFP-GmGγ6 and YFP-GmGγ7 also showed fluorescence restricted to the periphery which suggests the predominantly plasma membrane localization for these proteins (Figure 6). This is intriguing as these proteins lack a canonical prenylation motif and do not have any cysteine residues in the vicinity for such modifications. This is similar to the localization of rice RGG2 protein to the plasma membrane despite lacking a prenylation motif at its C terminal [21].

GmGγ8 and GmGγ9 have a conserved C at position 4 and all three group III genes have a conserved cysteine at position 6 from C-terminal in addition to multiple other cysteine residues in the vicinity. These cysteines qualify for the possible lipid modification by farnesyl transferase or by gernylgernyl transferases [35]. However, the localization of this group of proteins is interesting as in addition to most of the protein being present at the periphery, fluorescence was also observed as clear puncta especially in the case of YFP-GmGγ8 (Figure 6). Such patterns are indicative of either protein aggregate formation or endosomal localizations [34]. Further studies with stably transformed plants will be required to critically assess the localization of group III GmGγ proteins.

### Protein-protein interaction between GmGβ and GmGγ proteins

**Figure 7:**
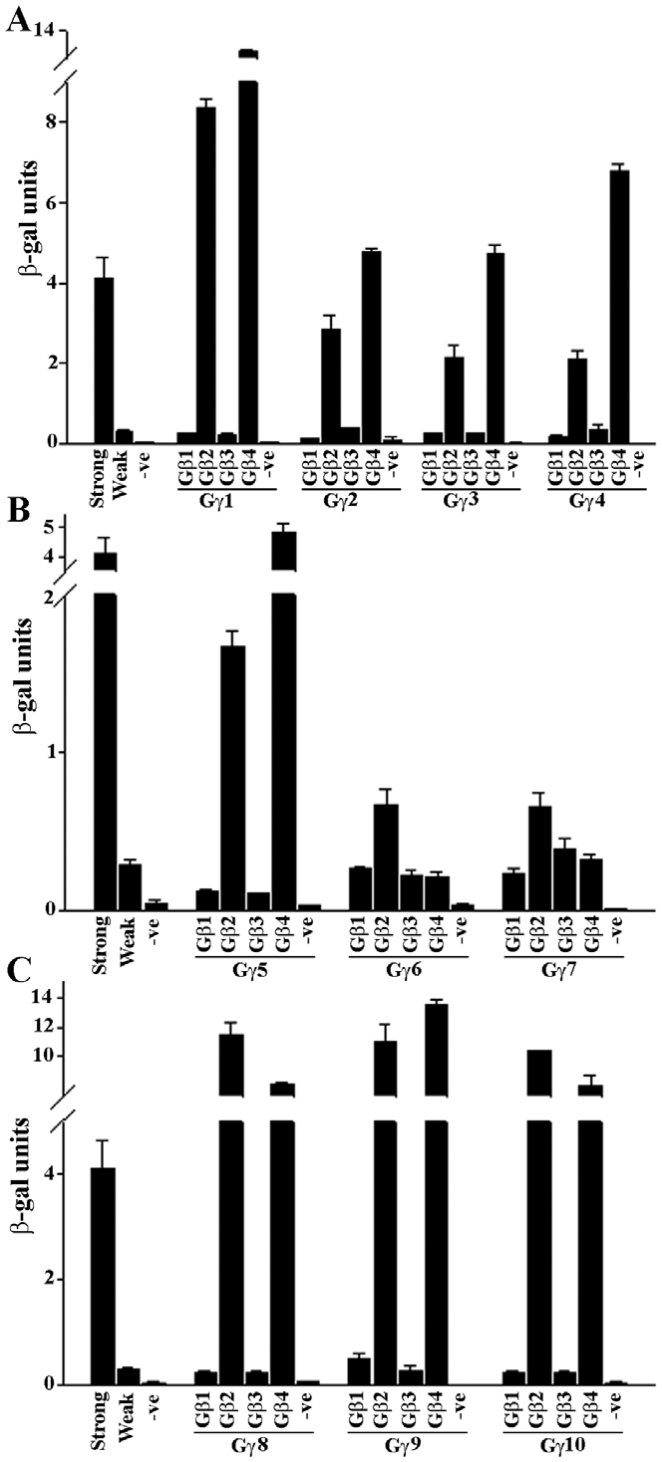
Interaction between soybean G-protein β and γ subunits. Interaction between GmGβ (in pDEST32) and GmGγ (in pDEST22) proteins was determined using yeast-2 hybrid-based colorimetric assay. The assays were performed in triplicates and data were averaged. Error bars represent the standard error of the mean. Two biological replicates of the experiment were performed with similar results. (A) Interaction between GmGβ proteins and group I GmGγ proteins. (B) Interaction between GmGβ proteins and group II GmGγ proteins. (C) Interaction between GmGβ proteins and group III GmGγ proteins. Strong, weak and -ve refer to the interaction strength between RalGDS-wt-pDEST32 with Krev1-pDEST22, RalGDS-m1-pDEST32 with Krev1-pDEST22 and RalGDS-m2-pDEST32 with Krev1-pDEST22, respectively The controls are provided with the ProQuest two hybrid system (Invitrogen).

We assessed the protein-protein interaction specificity of the three groups of Gγ proteins with all four Gβ proteins of soybean using the ProQuest yeast-2-hybrid system. The group I proteins GmGγ3 and GmGγ4 showed strong and specific interactions with GmGβ2 and GmGβ4 but not with GmGβ1 and GmGβ3 proteins (Figure 7A) as is also the case with GmGγ1 and GmGγ2 [10]. The group II GmGγ proteins exhibit relatively weaker interaction with the GmGβ proteins compared to the group I proteins except the interaction between GmGγ5 and GmGβ4. However, specific interactions of group II proteins were observed with all four GmGβ proteins (Figure 7B).

The establishment of interaction with Gβ proteins was an utmost requirement to classify the group III proteins as novel Gγ proteins. As shown in Figure 7C, the group III GmGγ proteins showed very strong and specific interaction with GmGβ proteins. Similar to the group I proteins, the group III proteins also interacted strongly with GmGβ2 and GmGβ4. However, distinct from the group I proteins, where no interaction was seen with GmGβ1 and GmGβ3, the group III proteins clearly show weak but specific interaction with GmGβ1 and GmGβ3 proteins (Figure 7C). We also tested whether the N terminus itself was sufficient for this interaction by making deletion constructs of proteins that either expressed the N-terminal half (till DPLL/DPFT motif) or the C-terminal half (protein sequence following the DPLL/DPFT motif). Significantly weaker interaction was detected using either of the truncated proteins (Figure S8), suggesting a full length protein is required for such interactions.

**Figure 8:**
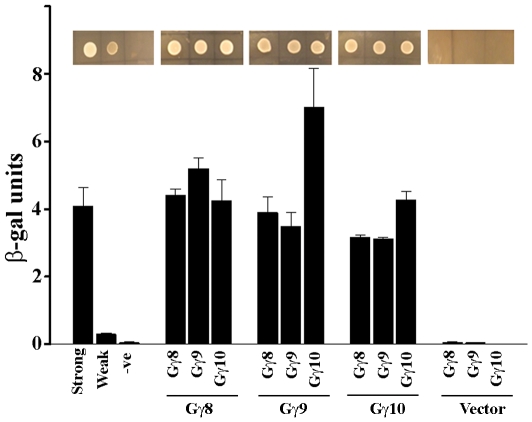
Interaction between group III GmGγ proteins. Interaction between different members of group III GmGγ proteins was determined using yeast-2 hybrid-based growth and colorimetric assay. The assays were performed in triplicates and data were averaged. Error bars represent the standard error of the mean. Two biological replicates of the experiment were performed with similar results. Inset shows growth of yeast colonies on media lacking Leu and Trp but containing 50 µM 3-AT. Strong, weak and -ve refer to the interaction strength between RalGDS-wt-pDEST32 with Krev1-pDEST22, RalGDS-m1-pDEST32 with Krev1-pDEST22 and RalGDS-m2-pDEST32 with Krev1-pDEST22, respectively.

The presence of long cysteine rich C-terminal regions on the group III GmGγ proteins prompted us to test whether these proteins interact with one another. We tested interaction between GmGγ8, GmGγ9 and GmGγ10 in all nine possible combinations. The proteins exhibit strong interaction with each other as shown in Figure 8.

## Discussion

### Identification of three distinct Gγ protein families in soybean

Gγ proteins are an integral part of the G-protein heterotrimer. In mammalian systems, these proteins are relatively small (7–8.5 kDa) and are the most diverse of the three subunits with only ∼50% sequence homology between different isoforms. The proteins also show a high degree of tissue specific expression and isoform-specific interactions with Gβ proteins. In both plants and animals, the Gγ proteins are required for proper targeting of the Gβ subunit and of the intact heterotrimer to the plasma membrane [5], [36]–[38]. Identification of diverse Gγ proteins from soybean (Figure 1) along with the presence of multiple Gα and Gβ proteins expands the number of possible heterotrimeric combinations in soybean in addition to identifying novel, plant specific components of G-protein signaling.

The C-terminus of Gγ proteins is the basis of our classification of the proteins into three distinct groups. Group I is comprised of canonical Gγ protein that have all the conserved features of Gγ proteins as described based on the mammalian paradigm. Homologs of this family are present in all plant species including gymnosperms and mosses. Most of the reported plant Gγ proteins to date, AGG1, AGG2, RGG1 and GmGγ1-4, are members of this group (Figure 1). The group II proteins consisting of Gγ5, Gγ6 and Gγ7 differ from the family I protein mainly due to the absence of conserved cysteines in the C-terminal region. The genes seem to have evolved from a single amino acid substitution of the CWIL motif to the RWI motif (the most common C-terminal motif present in dicot plants) as most of the protein sequence and exon-intron organization of family I and family II proteins is highly conserved (Figure 2). The rice RGG2 protein is a member of this family although the protein ends in a KGDFS sequence which also seems to be conserved in other monocot species. Lack of cysteine residues in group II proteins precludes the possibility of prenylation; however, the proteins do seem to localize at the plasma membrane (Figure 6) as has also been reported for the rice RGG2 (21). A single cysteine present in the middle of these proteins could be a potential target for palmotylation which might assist in its anchoring to the plasma membrane [21]. Additionally the proteins have a high number of positively charged and aromatic amino acids at the C-terminus (7 out of 10) which may target them to the plasma membrane by the formation of an α helix [39]. In mammals, lack of prenylation either by mutation of the conserved cysteine residue or by chemical inhibition has been shown to result in localization of specific Gβγ proteins to the nucleus of the cells and its possible role in regulating transcription, a function not typically associated with G-proteins [40]. Lack of a prenylation-less gene in the *Arabidopsis* genome has limited the functional characterization of this family of Gγ in plants. The availability of an insertional mutant line in RGG2 might be able to resolve the issue of whether such proteins play any unconventional roles in plants.

The group III proteins constitute a novel Gγ family, specific to plants. Homologs of these proteins are present in both angiosperm and gymnosperm families. We applied the following criteria to establish the group III proteins as authentic Gγ proteins. The coiled-coil domain of group III Gγ proteins is highly similar to the conventional Gγ proteins with full conservation of amino acid residues involved in the interaction with Gβ proteins (Figure 1). The size of the second and third exons of these proteins is very similar to group I and group II Gγ proteins (Figure 2). Additionally a homology modeling-based analysis of three-dimensional protein structures using a fold recognition server Phyre (**P**rotein **H**omology/analog**Y** **R**ecognition **E**ngine, http://www.sbg.bio.ic.ac.uk/~phyre/) predicted these proteins to be Gγ proteins with 40–55% precision. Finally the proteins showed strong and specific interaction with the GmGβ proteins. The *Arabidopsis* homolog of group III family protein At5g20635 has recently been identified as a novel Gγ protein [23].

Rice has two proteins that show homology to group III proteins, *DEP1* (dense and erect panicle 1) and *GS3* (grain size 3), that have been isolated as major QTLs for seed size and yield [24], [25]. Interestingly, the rice Gα protein RGA1 is also involved in regulation of seed size [41]. The rice DEP1 and GS3 proteins have been described as novel proteins containing a TNFR motif, a transmembrane domain and proteins with homology to human keratin-associated protein. We also identified a TNFR motif in the soybean group III family proteins; however, using multiple transmembrane prediction programs including TMHMM (http://www.cbs.dtu.dk/services/TMHMM/), HMMTOP (http://www.enzim.hu/hmmtop/) and DAS (http://www.sbc.su.se/~miklos/DAS/), we did not identify any transmembrane domains in the GmGγ8, GmGγ9 or GmGγ10 proteins. Rice and *Arabidopsis* group III proteins are predicted to have a single transmembrane domain using DAS, but not with TMHMM or HMMTOP. Experimental verification of the presence of a transmembrane domain and any possible role it might play in localization and/or positioning of these at the plasma membrane will be needed to evaluate its importance. Interestingly, YFP-fused group III GmGγ proteins, in addition to the peripheral YFP fluorescence, also showed small vesicle like structures which were very evident in GmGγ8. These proteins might be localized to endosomes structures in addition to the plasma membrane. However, since these proteins are highly cysteine rich such structures could also be due to protein aggregate formation or self-interaction (Figure 8). Our data at this time cannot differentiate between these possibilities. Expression of proteins with native promoters in a protein null background will help decipher correct localization.

### Expression profile of soybean Gγ proteins and possible correlation with Gβ proteins

The analysis of the complete repertoire of the *GmGγ* genes and its comparison with the expression pattern of *GmGα* and *GmGβ* genes began to display specific expression patterns related to particular genes or gene families. Moreover, when comparing the absolute expression levels within different subunits, a wide range of expression levels were observed for the *GmGγ* genes (e.g. Figure 3, *GmGγ9* versus *GmGγ6*), whereas all *GmGα* and *GmGβ* genes were expressed at a relatively similar level to each other. Additionally the duplicated gene pairs of *GmGα* or *GmGβ* typically showed similar expression patterns, a trend not observed between duplicated gene pairs of *GmGγ* genes. *GmGγ9* was the most highly expressed gene, whereas its duplicated gene *GmGγ8* was expressed at the moderate level. Likewise, *GmGγ4* was a highly expressed gene but its duplicated gene pair *GmGγ3* was relatively poorly expressed.

Some tissue specificity of gene expression was also evident while comparing the expression of multiple *Gγ* genes such as low expression of group II genes in reproductive organs or lower expression of group III genes in nodules (Figure 3). Additionally, during seed development and germination, specific expression profiles were observed for individual genes which in some cases corresponded well to the expression of *GmGβ* genes. These observations suggest that developmental stage-specific or tissue-specific expression of particular genes may lead to specific βγ combinations, similar to what is observed in the mammalian systems [42], [43].

Since the two rice homologs of group III genes DEP1 and GS3 are involved in grain size determination and yield, we focused on the expression pattern of soybean group III *GmGγ* genes during seed development. Our data showed that of the three group III genes in soybean, *GmGγ8* shows the most interesting expression pattern during seed development and germination (Figure 4, 5). The expression of this gene was highly up-regulated when seed is undergoing maturation (stages S7 onwards), whereas a sharp decrease was observed during seed germination. This gene could be a true functional homolog of the rice *DEP1* or *GS3* gene. Additionally the expression of this gene in seeds could be dependent on the endogenous ABA and/or GA concentration as the levels of both these hormones change significantly during seed maturation and germination. Interestingly, *GmGγ5* also followed a similar expression profile where the expression was up-regulated during seed maturation and generally down-regulated during germination. These genes could be potential targets for manipulation to regulate soybean seed development. It was also obvious that this group of genes is highly variable functionally as *GmGγ10* shows very little expression in seeds at all developmental stages and exhibits no change in expression profile during germination.

### Interaction specificity of GmGβ and GmGγ proteins

Specific mammalian Gβ and Gγ proteins form non-dissociable dimers and interact very strongly under a variety of *in vitro* and *in vivo* conditions. The data presented in this study suggest that there is specificity of interaction between different GmGβ and GmGγ proteins. It is especially intriguing that the GmGβ1 and GmGβ3 are in general weaker interactors compared to GmGβ2 and GmGβ4 even though they have more than 90% sequence similarity at the protein level (Figure 7). Additionally the group II GmGγ proteins also exhibited weak interactions compared to the group I and group III proteins even though they do interact with similar strengths with all four GmGβ proteins. Similar differences in the interaction between mammalian Gβ and Gγ proteins have also been observed. The human Gβ1-4 share 80–90% sequence identity; however, Gβ1 in general interacts with multiple Gγ isoforms, Gβ2 is more restricted in its interaction partners and Gβ3 displays significantly weaker interactions [44]–[46]. In most cases, however, the interaction data were based on *in vitro* assays and its relevance in the context of a specific cell type or a signal remains to be evaluated in both mammalian and plant systems.

An intriguing observation in our studies is the strong interaction between different members of the group III proteins themselves (Figure 8). It would be interesting to assess how the oligomerization of these proteins might affect interaction with GmGβ proteins or other possible interactors. The unusual nature of these proteins does not preclude the possibility of its involvement in some plant-specific signaling mechanisms which are different from what is known from studies in mammalian systems. Plants do have several unconventional G-protein components such as the extra-large G-proteins that have a Gα domain [47]–[50]; the GTG proteins that have GTP-binding and hydrolysis activity of their own and are regulated by GPA1 [51]; and the RGS1 protein that has a 7TM GPCR-like structure fused to RGS domain [52]. Likewise, most of the known effector proteins of G-protein signaling in plants are also distinct from the conventional effector proteins of mammalian systems. For example, PRN1 (Pirin1) which is a member of an iron-containing subgroup of the cupin superfamily, PD1 (prephenate dehydratase1), a protein involved in phenylalanine biosynthesis, and a NF-Y family transcription factor form a signaling complex during G-protein mediated light and ABA signaling pathways during early growth and development in *Arabidopsis* [53]–[55]. Similarly, a chloroplast-localized protein THF1 (thylakoid formation 1) is a GPA1 effector protein during sugar signaling [56]. Detailed study of specific pathways mediated by these unconventional proteins in the context of canonical heterotrimeric G-protein signaling is only in its infancy and future work may divulge additional signaling mechanisms specifically evolved in plants.

### Conclusion

We have identified three distinct families of Gγ proteins including a novel, plant-specific Gγ protein family in the soybean genome. The elucidation of this complete repertoire of different G-protein subunits in soybean reveals a highly elaborate G-protein signaling network in plants. Our data also suggest the presence of subunit-specific and tissue-type or developmental stage-specific heterotrimeric combinations. Additionally the homologs of the group III Gγ protein have been identified as major QTLs for grain size and yield in rice. Further work with the generation of RNAi and overexpression lines of soybean G-protein genes will help us decipher its signaling mechanisms as well as its use as potential targets for biotechnological applications.

## Materials and Methods

### Plant material and growth conditions

Soybean (*Glycine max* L.) cv. Jack seeds were grown in growth chamber (26/20°C day/night temperature, photoperiod of 14/10 h, 800 µmol m^−2^ s^−1^ light intensity, and 60% humidity). Different developmental stages of soybean plants were collected, immediately frozen in liquid nitrogen and stored at −80°C. Tissue for germination and stress-related experiments was prepared as described in [10].

### Cloning of soybean G-protein genes

Soybean Gγ genes were identified by analysis of the latest the soybean genome assembly (www.phytozome.net/soybean) with *Arabidopsis* and rice full length and middle coiled-coil region Gγ protein sequences as queries. Full-length Gγ genes were amplified from soybean seedling cDNA using gene-specific primers (Table S1). The eight newly identified genes were cloned into the pENTR/D-TOPO vector and confirmed by sequencing.

### RNA isolation and qRT-PCR

Total RNA was isolated from different tissues of soybean plants using Trizol reagent (Invitrogen) and qRT-PCR experiments were performed essentially according to [10]. The real-time PCR amplification was repeated three times and data were averaged. Sequencing and melt curve analysis of amplicons confirmed specificity. Standard curves for each of the genes were generated using the cloned plasmid DNA of each gene.

### Localization of GmGγ proteins

The ten *GmGγ* genes were cloned into the pEarleyGate 104 [57] destination vector using LR clonase mix (Invitrogen). Sequence-confirmed recombinant plasmids containing the *YFP*::*GmGγ1-10* were transformed into *A. tumefaciens* strain GV3101 for subsequent plant transformation.

Abaxial surface of tobacco leaves were infiltrated with a log-phase culture of *A. tumefaciens* containing either the gene of interest or an empty vector control according to [58]. Infiltrated plants were incubated in darkness for 36 h followed by 24 h in light. The leaves were imaged with the Zeiss LSM 510 laser scanning confocal microscope (Carl Zeiss) using a 40x water-immersion, 1.2 numerical aperture, C-Apochromat objective. The yellow fluorescent protein (YFP) was excited with the 458-nm line of the argon laser. At least three independent infiltrations were performed for each construct.

### Protein-protein interaction assays

To determine the interaction between specific GmGβ and GmGγ proteins, GATEWAY-based yeast-two-hybrid assay was performed (ProQuest Two Hybrid System, Invitrogen). Briefly, *GmGβ1-4* genes were cloned into pDEST32 bait vector (containing DNA-binding domain) and *GmGγ1-10* genes (full length, N-terminal and C terminal parts) were cloned into pDEST22 prey vector (containing DNA-activating domain). The constructs were co-transformed in yeast host strain MaV203 (Invitrogen) in specific combinations. Interaction was determined by growth of diploid yeast colonies on minimal media lacking leucine and tryptophan, but containing 50 µM 3AT (3-Amino-1,2,4-triazole). The quantitative strength of interaction was determined by β-galactosidase (β-gal) expression assay using ONPG (o-nitrophenyl-β-D-galactopyranoside) as a substrate per manufacturer's instructions.

## Supporting Information

Table S1 **Gene-specific primers used for expression analysis of** ***GmGγ*** **genes.**
(DOCX)

Table S2 **Absolute copy number quantification and primer amplification efficiency test of** ***GmGγ*** **genes.**
(DOCX)

Figure S1 **Genomic sequence of the newly annotated** ***GmGγ3*** **on chromosome 20.**
(DOC)

Figure S2 **Genomic sequence of the newly annotated** ***GmGγ4*** **on chromosome 10.**
(DOC)

Figure S3 **Correct genomic sequence of** ***GmGγ5*** **as experimentally verified.**
(DOC)

Figure S4 **Correct genomic sequence of** ***GmGγ8*** **as experimentally verified.**
(DOC)

Figure S5 **Correct genomic sequence of** ***GmGγ10*** **as experimentally verified.**
(DOC)

Figure S6 **Evolutionary relationships of GmGγ proteins.**
(PPT)

Figure S7 **PCR efficiency of** ***GmGγ*** **genes over 100,000 fold dilution.**
(PPT)

Figure S8 **Test of interaction between the N-terminal and C-terminal parts of GmGγ 8, 9 and 10 proteins with different GmGβ proteins.**
(PPT)
