# Supplementary material for: Conventional and Novel Gγ Protein Families Constitute the Heterotrimeric G-Protein Signaling Network in Soybean
Source: PLoS One. 2011 Aug 10;6(8):e23361. doi: 10.1371/journal.pone.0023361 (PMC3154445; doi:10.1371/journal.pone.0023361)
Supplement: Table S1 — Gene-specific primers used for expression analysis of GmGγ genes. (DOCX) [file pone.0023361.s001.docx]

**Table S1. Gene specific primers used for expression analysis of *GmGγ* genes.**

| **A. Primers used for G-protein gene amplifications** |
| --- |
| 1. GmGγ1 DTOPO FP 5′ CACCATGGCGTCTGAAACGGCGT 3′ |
| 2. GmGγ1 DTOPO RP (S) 5′ TCAAAGTATCCAACATCTACAT 3′ |
| 3. GmGγ2 DTOPO FP 5′ CACCATGGCGTCTGAAACGGCGT 3′ |
| 4. GmGγ2 DTOPO RP (S) 5′ TCAAAGTATCCAACATCTAC 3′ |
| 5. GmGγ3 DTOPO FP 5′ CACCATGGAATCCGGTGGGCCTGAATC 3′ |
| 6. GmGγ3 DTOPO RP (S) 5′ TCAGAGAATCCAGCATCTACAGCA 3′ |
| 7. GmGγ4 DTOPO FP 5′ CACCATGGAATCCGGTGGGCCTGAATCCGCA 3′ |
| 8. GmGγ4 DTOPO RP (S) 5′ TCAGAGAATCCAGCATCTACAGCAGCT 3′ |
| 9. GmGγ5 UTR FP 5′ CTAAGTGGTGTGATGATAGCCATGGA 3′ |
| 10. GmGγ5 DTOPO FP 5′ CACCATGATAGCCATGGATGGACACCAACC 3′ |
| 11. GmGγ6 DTOPO FP 5′ CACCATGATAGCCATGGATGGACAC 3′ |
| 12. GmGγ7 DTOPO FP 5′ CACCATGATGGCCATGGAAGGACAA 3′ |
| 13. GmGγ5/6/7 DTOPO RP (S) 5′ CTAAATCCAACGTTTGTGATTTCG 3′ |
| 14. GmGγ8 DTOPO FP 5′ CACCATGAGCACTCCCACAAGAGGCACT 3′ |
| 15. GmGγ8 DTOPO RP (S) 5′ CTAGAAACATGAACAAGGGT 3′ |
| 16. GmGγ9 DTOPO FP 5′ CACCATGGCCACTACTCCCACCACCGT 3′ |
| 17. GmGγ9 DTOPO RP (S) 5′ CTAGCAACAAAAACAACATGGTCT 3′ |
| 18. GmGγ10 DTOPO FP 5′ CACCATGGATGGTGGTGGCTACAA 3′ |
| 19. GmGγ10 DTOPO RP (S) 5′ CTAGCAACAATTTTGAGCACA 3′ |

| **B. Primers used for qRT-PCR** |
| --- |
| 20. GmGγ1 RT FP 5′ GGGTGCAGGAGGAACTGATA 3′ |
| 21. GmGγ1 RT RP 5′ ATCTTGAGGCCCTTCAAACC 3′ |
| 22. GmGγ2 RT FP 5′ GGTGCAGGAGGGACTGATAA 3′ |
| 23. GmGγ2 RT RP 5′ ATCTTGAGGCCCTTCAAACC 3′ |
| 24. GmGγ3 RT FP 5′ CGCTTGGAGCAAGAAGCACGA 3′ |
| 25. GmGγ3 RT RP 5′ CTTCAAACCACCGATCCCATGT 3′ |
| 26. GmGγ4 RT FP 5′ ATGGAATCCGGTGGGCCTGAATCCGCA 3′ |
| 27. GmGγ4 RT RP 5′ CTTCAAACCATCGATCCCATGC 3′ |
| 28. GmGγ5 RT FP 5′ ATGGAGAAGAAGGGAGTGAAAAG 3′ |
| 29. GmGγ5 RT RP 5′ TCGATCCCAACCAGCATCAACTGAACC 3′ |
| 30. GmGγ6 RT FP 5′ GAGAAGAAGGGAGAGAAAAGG 3′ |
| 31. GmGγ6 RT RP 5′ CCAACCAGCATCAACTGAACT 3′ |
| 32. GmGγ7 RT FP 5′ TTAGTATTTTGCAGGAAGAGTTGA 3′ |
| 33. GmGγ7 RT RP 5′ ATCGATCCCAACCCGCATCG 3′ |
| 34. GmGγ8 RT FP 5′ AAGATTCAGATGCTGGAAAGAGAG 3′ |
| 35. GmGγ8 RT RP 5′ ACATGACTGGTGGTTCTTCTTGCT 3′ |
| 36. GmGγ9 RT FP 5′ CATGCTCGAGAGGGAAATCACT 3′ |
| 37. GmGγ9 RT RP 5′ GTTAAAACAGGGCATGCCACAG 3′ |
| 38. GmGγ10 RT FP 5′ ATTTTCATGGGAAGCGGAAGCA 3′ |
| 39. GmGγ10 RT RP 5′ TAGAGACTGTCTGTTTTCTTGTG 3′ |

| **C. Primers used for amplification for yeast-2 hybrid-based interaction tests** |
| --- |
| 40. GmGγ8 N-ter RP 5′ CTTCCACAAGCTACATGACTGGTGGT 3′ |
| 41. GmGγ9 N-ter RP 5′ GAGCCACTTCCAGAAGCGACATGACC 3′ |
| 42. GmGγ10 N-ter RP 5′ CCTCCTGAAGTGATGGGATTTAGAGA 3′ |
| 43. GmGγ8 C-ter FP 5′ CACCTGTGACATCCCTTGCTTTAACTTGT 3′ |
| 44. GmGγ9 C-ter FP 5′ CACCTGTGGCATGCCCTGTTTTAACCTCT 3′ |
| 45. GmGγ10 C-ter FP 5′ CACCCAGATGATCAGTCTTCCACGGGTTT 3′ |
