# Supplementary material for: Conventional and Novel Gγ Protein Families Constitute the Heterotrimeric G-Protein Signaling Network in Soybean
Source: PLoS One. 2011 Aug 10;6(8):e23361. doi: 10.1371/journal.pone.0023361 (PMC3154445; doi:10.1371/journal.pone.0023361)
Supplement: Table S2 — Absolute copy number quantification and primer amplification efficiency test of GmGγ genes. (DOCX) [file pone.0023361.s002.docx]

**Table S2: Primer amplification efficiency test of soybean G-protein genes.** Plasmid DNA (G-protein gene in pENTR:D-Topo vector) was used to test the primer amplification efficiency and calculate the correlation coefficient over 100,000 fold of serial dilutions. The primers were tested on cDNA sample from primary root tissue, used as the reference tissue for all qRT-PCR analysis. Each value represents the average Ct value of two independent experiments.

| **Target** | **Plasmid**  **(1e+5)** | **Plasmid**  **(1e+3)** | **Plasmid**  **(1e+1)** | **Correlation coefficient**  **( R^2^)** | **cDNA (Root)** |
| --- | --- | --- | --- | --- | --- |
| **GmGγ1** | **8.814** | **15.908** | **22.808** | **0.999** | **26.890** |
| **GmGγ2** | **8.944** | **15.987** | **23.019** | **1.000** | **27.802** |
| **GmGγ3** | **7.987** | **15.638** | **22.446** | **0.999** | **26.785** |
| **GmGγ4** | **8.310** | **15.904** | **22.634** | **1.000** | **26.062** |
| **GmGγ5** | **9.842** | **16.928** | **23.772** | **0.999** | **26.380** |
| **GmGγ6** | **9.181** | **16.273** | **22.830** | **0.998** | **28.699** |
| **GmGγ7** | **9.793** | **16.925** | **22.528** | **0.998** | **26.211** |
| **GmGγ8** | **9.514** | **16.009** | **21.981** | **0.999** | **28.276** |
| **GmGγ9** | **9.589** | **16.702** | **23.876** | **0.995** | **25.376** |
| **GmGγ10** | **9.149** | **15.970** | **22.769** | **1.000** | **29.170** |
