# Supplementary material for: Conventional and Novel Gγ Protein Families Constitute the Heterotrimeric G-Protein Signaling Network in Soybean
Source: PLoS One. 2011 Aug 10;6(8):e23361. doi: 10.1371/journal.pone.0023361 (PMC3154445; doi:10.1371/journal.pone.0023361)
Supplement: Figure S1 — Genomic sequence of the newly annotated GmGγ3 on chromosome 20. (DOC) [file pone.0023361.s003.doc]

**Figure S1.**

**GmGγ3 (Gm20g33310.1)**

**ATGGAATCCGGTGGGCCTGAATCCGCAAGCCCCATGACCCACAGGGTTCAGTCTCTGTCTTCCGCAGATACAAGAGGGAAACATAGGATACATGCTGAACTCAAACGCTTGGAGCAAGAAGCACGATTCTTAGAGG**TACGTACTCTTTTTTTTCTTCTCTTCATAATTTATATCTTGGGGTTTTGTTCATTGGTGGGGTGGTGATTTTTTTCTGTTTGTTTTTGATTGTTCTCTGTTTGTGTCGTTTTTTCCATTGGCCTTTTGCTTTGCTTTTTGATTGGTTTACTTTTTTTTTTTTGGTTCTTCTCAACGAGAGATGGTAAAATGAGTTTGAGAAAGGTTTTAACTTTTCTTTGTTGGTGTGTCGGTATTATCAGAGACAAATGCTATCTGTGAACCTGATGACGTTGCCTACAAATTAACAACAACAAAAAATTATTTGGTTGATTGCTGGGGTATTCTTCTGTCAGCTTGATTTATGAGTTTTTGTTTGTGGAAACACTGTTTAAGAAATTAATGTGCAGATTGGAGAGGTACATGTTATTGTGGTCCGTGAACCTCAACTATAGTGTGGCCTCCTGATAGTTAAAAGGGTAGTTATTTTATTAAATGCATGGTTTTCCAAAATATACACTTACATTTACACCAATTTGGTCCTACAAAGATTTTTGAATTCCAATCAGTCCTCTGAATATTTAAATTGACATGAAAATGGTTCAAGTTTGTGTCAATTAGCTAGACAAAAGGATTGATTTGGTGTATTTTTTAATCTAAGGGTTCCTGATTTGATGTTGCATATCTTTAGAGGATCAGATTGATGTCAAGTGTATCTTTGAAGGACCAAACTACTGGTAGTTTGGTTATTGTTTCCCCAATGAATATTTTCTGGATATCATAATTAATGTTTTGATATATTAATTGTTTACCTGTTAAAAAGGCGTTTGGGTTTTGATTATCAATTATTCTTAATGAACAAAATGCGATTTTAACTGGCAAAGTTATCTTTAGATGTGGAAACATTTCAGTTGAAGATATCAACCGTTCCTGAGTCTTTGCTTAGGAAGCTTCACTTTTCTAAGATTAATAGATTAATCTGTCTATATGCACTGTCAATCTGTCATTACAGCGACCAAAGCTTAATGATTGAGATAGTATAATTAAACCGATGTTGTTTTTTTTACTTTAATTTTTGTCGTTTCTTGCTTACTCATGTCGTAAGTTGCGGTTTTTGGACAATTTTGCATTTTCATTTTGTAGGAAACTGCCAGAAAGTTCATTTAGTTACACTCAGGGGCACAGTTTTTCTTTCAGATGACTCTAAATTTACAATCTACTTTATCACTCTTTTTACTAGTGGCTGCTTTACTTAAAACAGCGATAGAATTTTAAAAACCTTATATAATTCATAATTGTAATAATGCAAAAATGGAATATGCACCTTGACTTTTGAAGGGATTTGCTGAAACTCAGCTGCTGCGTTTCAAATATTTGGAGAGACATCTATTGAGCAAGGGAGAGAGACTGAGATCTTTCACTTGATAGTTGTATCAAAGGTTCAGCGTTGTATTGCATGTTGGGAACCTGGGGAGAAAGAATCACCTTTAGAATGTGAAAACTATAACTATTAGCTTCTTAAAATCACATCCAAGATATAGAACCAAACATGCCTTTAAAAACTTTATCCAGGTTACCATCACTCATAACTGTCTAACAACAAAGATTGCCGGTTGAAAATTTTATGAAATTTTCTATATTAAAATATCAGGTGATTTTTCTAAATGTTTATTTATGCACAAACAAGTTTATCAATGGTGGGTAACCAAAATTCTGCCATATAATCACACCATTGTGGCATATGGCGCCGCCATAGTGGGCTTCCCTTCATAAATTGCCTATGGCAAAGGGGTTGAATGCCTTGTTATAGTGTTATGGCGACACTATGATGGTTATTTTAACAACATTGCGCACAAATCAACTTTAGCATTTTCTGTTTATGGGACAAAGTTGGCCCTGCAGGGATTGTGGTAGTGTTTTTGTTTTTGATAATATTTTTGTAGTCAACAAAAATAAACAAGTTGGAGAAATTTAGCTAGTCCACCTCCTTTTAGATATAATTGCCGTTGACATAGTTGGCTTAATATATGAATGTACCTTCTTGGGTTATTTTCTGATGAGTGTTTACTGGAACTGGGGGGTAATAATTTCATGAAGTAGGACCTCCATCATTCCAAAATAAACCTTGCTGTTTGCATTTGCTTCTCATTTATCAATAATTTATTATTTCCCCACTGTTTTGATTCACTTTGTACCGTTATAAGCAATTAAGCATCAATTACTCAAATGGTTCAACCCTGTTTTTGGTAATAATTGAGATTTGAGATTCCATTTCTCAACTAGCAATCTTTAAAACATGGGACAGACCACAGAGGGAATATGATTTATCACCAAAATCCTTCCAATTCTTTGGTTCCTCTTTTTCTATGTGAGCTGGCGAAGGTATAGATAATGAATTGTAAGTTCTATGACATATTAGAAACCAAAATTTGATATTTTAGATCACTTACAAGAAGGTATGGCGTATTGAATTGGGGAATGGAGAGTATGCTCTAAGAACTTGATTTAGATTTGTTGCCTTCATTTATCCTTACCAACCTAAAAGATTCAAATTTAGGTGTGAAGGAAATTTAGAAACATCAAATTGGGGGTTTGTCAAACTGGATAGATCAGTGCCAACACCCTTTTGAGCTCCTGTTAAGGATATGCAATTAAGCAATCATATCATTGGATGTTCGGAGAACTAGCAAAATCCTGTGCTATTAACCTTATCGGCATCAAGGTTACTAGGAGTAATTAAGCAATCATATAATGTTGCAAAGGTCATTCACTGCCCCCTCCTTTGTCTCTTTCGGTGATTTGCCAAAGACCAGTGAAGTTGATAAGGGGGCCACTTGTAACTTTTTAGTGATCATCTCTGTCAAATATTATTATCTTGTTTAATAACAGTTTCATGATCTGGAATATGTTATCAAGTCCTTACCCAGTTAAGATTATTTGGTAATCCATCATGAGGCCCACTTCATCCATGGAGGAGAGAAAGAAGCAACCATTAATTTCTTAGATATATAGGTTGATTTTTTTTTAATGCTTTAAGTGGCCAAGGCCAACTATGTAAATCCGTTCTATAAGTGGGTAGCACCGTTGGACAGGGAGGATTAAATATTTTTTGGACTACATACCTGTGAAACTGCTTCCTTCTCTGTTTATTATATATCCCTCATAGTTGGCATAAGCTCATTGTTAGGAGTCTTGTTATTGGTTTTATTATTTTCCGTTTGTATCTTAATCTTTAGGGAGATGGGATATGAAAATTCTAAGGACATGAAAACTCTTTCATGCTTATTTTTTGAGGTACAAGTTTCAGAATACCCTGTGGAACTGAATGTTCTTTTCTGGTCAGGATGACTAGATATGGCTTCCCAAAGAGAAAGGGGAAGGCAATTGTAGTTGTCGCCCTTGACTAAAGGGTAGTCCAATACTCCAATCCTCTTCCATGTGGTCTTGTTTACATTGCCCTTTTCCTTTTTCTACATTTTTTCCCTAAGGCTAGGTGTGACCGAGTTCAGGAGGGGGATGGTAGGCATAGGATTCAGGTAAAGCAGCTTGCCCAGGATTCCATCCTGGGATCTAGCGCTTGCAGTAAGGGATTTGTGCCATTTGATCTATGCTGTTTGGCATGCACATTGCCTTTCAGTTTCACTATTATGATGCTCATATATTCACTATTTATGTAGGATTAAATTCCGTAGTCTTTGATGAAAATGTATGGGAGGTCTAAGAAGCCGTCTGGTGAGCTTTTCATAGGAAAGGCTGATTGTGGTTCTTAGCCTCCCTTATCAATTACCCGTATTTAGTTCTAGTTTTCAATACTCAATAGTCACTATAAAATGAATGCAGG**AAGAGCTGGAACAACTTGAAAGGATGGAGAAAGCATCTACATCGTGCAAAAT**GTAAGCTTAGAAGTAGAAAATGATTCGATAATGTATATAATTTGTGCTGGTTTAACTTTGAATTTCCATCCGGTTTTTGTTTTGCCAG**AATGCTCATCAATGTGGAAACAAAACCTGATCCATTACTACCATC**GTAAGATCTCTTAAGTGAACTTTTATTTACGTTGCAACCACGTGCTATCTTTGATGTGTAACAATTTTTTTTGGGGGGGTTTGACAG**ATCAGTTGGTCCCCTAAGTCCTACATGGGATCGGTGGTTTGAAGGCCCCCAAGATTCTAAAAGCTGCTGTAGATGCTGGATTCTCTGA**
