# Supplementary material for: Conventional and Novel Gγ Protein Families Constitute the Heterotrimeric G-Protein Signaling Network in Soybean
Source: PLoS One. 2011 Aug 10;6(8):e23361. doi: 10.1371/journal.pone.0023361 (PMC3154445; doi:10.1371/journal.pone.0023361)
Supplement: Figure S2 — Genomic sequence of the newly annotated GmGγ4 on chromosome 10. (DOC) [file pone.0023361.s004.doc]

**Figure S2.**

**GmGγ4 (Gm10g32215.1)**

**ATGGAATCCGGTGGGCCTGAATCCGCAAGCCCCATGACCCACAGGGTTCAGTCTCTGTCCTCAGCAGATACAAGAGGGAAACATAGGATACATGCTGAACTCAAACGCTTGGAGCAAGAAGCACGATTCTTAGAGG**TACGTACTCTTTTTTTTTCTTCTCTTAAAAATCATCATTATAATATCTTGGGGTTTTGTTCGTTGGTGGGGTGGTGATTTTTTTTTTCTGTTTGTTTGTTTATTTGTTCTTTTTTTGTGTCGTTTTTTTTTCTCTATTAGCCTTTGCTTTGCTTTTTGATTGGTGTACTTTTTTTTGTTCTTCTGAACGAGAGATGCTAAAATGAGTTTGGGAAAGGTTTTAACTTTTCTTTGTTGGTGTGTCGGTATTATCAGAGACAAATGCTATCTGTGAACCTGATGACGTTGCCTACAAATTAACAAAAAAAAAAAAATATTTGGTTGATTGCTGGTGTATTCTACTATTATTCTGTCAGCTTGATTTATGAGTTTTGTTTGTGGAAACACTGTTTAAGAAATTAATGTTCAGATTGGAGAAGTACAGTACATGTTATTGTGGTCCGTGATTCTCAACCATAGGGTGGCCTCCTTATAGTTAAAAGGGTAGTTGTTTTATTAAATGCATGGTTCTCAAAAGATACACTTACATTTACACCAATTTGGTCCTTCAAAGATTTTTGACAACCAATCAGTCCTCTGAATATTTAAATTGACATGAAAATGGTTCAAGTTGATCTCATATATAGCTAGACAAAAGAGTTGATTTTGTGTATTTTTTAATCTAAGGATTCCTAATTTGATGTTGCAAATCTTTGGAAGATCAGATTGATGTCAAGTGTATCTTTGGAGGACCAAACTGGTAGTTCAGTTATTGTTTCCCCAATGAATACATTCCAGTAATCATAATTAATGTTTTGATAAATTAATTGTTTACGCCGTTATAATGGTGTTTGAGTATGATTATCAGTTATTCTTGATGAACAAAATGCGATTTTAGCTGACAAAATTGTCTTTTGAAATATGTGTGAACATTTCAGTTAAAGATCCATCAACTACCGTTCCCAAGTCTTCGCTTGTCAACCTATCATTACAGTGGCCGAAGCTTAATGATTGATATAGTATAATTAAACTGTTGTTGTCTTTTTTACTTTAATTTGTGTCGTTTCTTGCTTACTCCTGTCGTAAGTTACGGTTTCGACAACTTTGCATTTTCATTTTGTAGGAAACTGCCAGAAAGTCCATTTAGTTGCACTCAGGCACCGTTTTTTCTTTGAGATGAATTCTAAATTTACAATTTACTTTATCACTCTTATTACTAGTGGCTACTTTATTTAAAACGGCAGTAGAATTTTAAAAATCATATGTGATTCATAATTGTAATTGTGCAAAAATGGAATATGCACCTTGACTTTTGAATGGATTTGTTGAAACTCAGCTGCTGCGTTTCAAAGGTTAGACGTTGTAAGACCTGATGAGAAGAATCACATTTGGAATATGAAAACTATAACTATTAGCTTCTTAAAATCGCATCCAAGATATGGAACCTAGCATGCTTTTAATAACTTTATCCAAGTTATCATGACTGATAACTGTCTAACAACAAATATTGCCGGTTGAAAATTTTATGAAGTTTTCTGTATTAAAATTTCAGTTGATTTTTCCAAATGTTTGTTTATGCACAAACAAATTTATCAATGTTGGTGGATAGCCAAAATTCCGCCCTATATACACACCATTATGGCATATGACGCCGCCATAGTGGGCTTCCCTTCATAGATTGCCTAAGGCAAAGGGGTTGAAAAATGTCACGTGATAGTGTTATGGCGGCACTAAGATGGTTATTTTAACAACAGTGTGCACAAATCAACTTTAGCATTTTCTGTTCATGGGAAAAAGTTAAACAAGTCGGAGAAATTTAGCTAGTCTACCTCCTTTTATATATAATTGCAATTGACATAGTTGGCTTAATATATGAATGTACCTTCTTTGTTAGGTTATTTTCTGATGACTGTTTATTGGAACTGGGGGATAATAATTTCATGAAGTAGGACATCCATTCCAAAATAAATCTTGTTGTTTGCATTTGCTTCTCATTTATCAATAGTTTATTACTTCCCTACTGTTTTGATTTACTTTGTACAGTTATAAGCAATTAAGCATCAATTACTCAAATATTTCAACCATGCTTTTGGAAATAATTGAGATTTGAGATTCCATTTCTCAACTAGCAATCTTTAAAACATGGGACAAACTACAGAGAGAATATGATTTTTCACCAAAATCCTTCCAATTCTTGGGTTCTTATTCTTTGATATGAGCCGGTGAAGGTATAGATAATGAATTGTAAGTTCTATGACAAATTAGAAACCAAAATTTGATATTTTAGATCACTTACAAGAAGGTTCTGCATATTGAATTGGGGAATGGAGAGAAGGCTCTAAGAACTTGATTTAGATTTGTTGCCTGCTTTTATCCTTACCAACCTAAAACATTCAAATTTAGGTGTGAAGGAAATTTAGAAACATCAAATTGAGGGTTTGTCAAACTGTATAGATCATTGCTAACACCTTTTTGAGCTCCTGTTCTGCTTGGTTAAGGATATGCAATTAAGCAATCATATCATTGAGTGTTCAGAGAACTAGCAAAATTCTGTGCTATTAACTTCATCGGCATCAAGGTTACTAGGAGTAATATAATGTTGCCGACATCATTCACTGTCCCCTCCTTTGGCTTTTTCGGTGATTTGCCAATGACCAATAGAGTTGATGAAGGGACGCTTCTAACTTTTTAGTGATCATTTGTCTCAAATATTATTATCTTGTTTAATAACAGTTTCATGATCTGGAATATGCTATCAAGTCCTTACCTGGTTAAGATTATTTGGTATTCATCATCTGGCCCACTTCATTCATGGAGAGGATAGAAAGAAGCAACCATTAATTTCTTAGATATATAGGTTGATTTTTTTGTTAATGCTTTAAGTGGTCAAGGTCAACTATGTAGATCTGTTCTTAAGTGGATTGCACCGTTGGACAGGGAGGATTAATTTTTATTTTTTTTACTATATACCTATGAAACTGCTTCCTTCTCTGTTTATATTATATCTCTCATAGTTGGCATAAGCTCATTGTTAGGAGTCTTGTATTGGTTTGATTATTTTCCCTCTGTATCTTAGTCTTTAGGGAGATGGGATATGAAAATTCCAAGGACTTGAAATCTCTTTCGTGCATATTTTTTGAAAGGTACAAGTTTCAAAATACCACGTGGAACTGAATATTGTTTTCTGATCAAGATGACTAGATATGGGTGAAATGGGAAGGTAATTGTAGTTGTAGCACTTGACCAAAGGGTAGTCCAATCCTCTTCCATGTGGTCTTGTTTACATTTCCCTTTTCCTTTTTCCACATTTTTTTCCCTATGGCTAGGTGTGACTGAGTTCAGGAGAGGGGTGGTAAGGCTAGGAATTAGGATGCAGGAAACGCAGCTTGCGCAGGATTCCATCCTGGGATCTAGAGCTTACAGTAAGGGATTTGTGCCATTTGATCTATGGCTGTTTGGCATGCACATTGCCTTTCAGTTTCAGTATTATTATGTTTGTATCTTCACTATTTCTGTAGGATTAAATTCCTTAGTCTTTGATGAAAATATATGGGAGGTCTAAGAAGCCATCTGGTGAGCTTTTCATAGGAAAGGCTGATTGTGGTTCTTAGGCTCTCTTATCCATGACTTGTATTTAGTTCTAGTTTTCAAATTTGGGTTGCAAGTGTTTCAATGCTCAATAGTCATTATAAAATGAATGCAGG**AAGAGTTGGAACAACTTGAAAAGACGGAGAAAGCATCTACAACGTGCAAAAT**GTAAGCTTAGAAGTAGAAAATGATTTGATATTGTTAATGTGTAATGTATATAATTTGTGTTGGTTTAACTTTGAATTTCCATCCGGTGTTTGTTTTGCCAG**AATGCCCAGCAATGTAGAAACAAAACCTGATCCATTACTACCATC**GTAAGATCTCTTATTTGAACTTCCTTTATTTATGCTGCAACCACGTGCTATCTTTGACGTGTAACAATTTTTTTGGGGGGGTGACAG**ATCAGTTGGTCCCCTAAGTCCTGCATGGGATCGATGGTTTGAAGGCCCCCAAGATTCTAAAAGCTGCTGTAGATGCTGGATTCTCTGA**
