# Supplementary material for: Conventional and Novel Gγ Protein Families Constitute the Heterotrimeric G-Protein Signaling Network in Soybean
Source: PLoS One. 2011 Aug 10;6(8):e23361. doi: 10.1371/journal.pone.0023361 (PMC3154445; doi:10.1371/journal.pone.0023361)
Supplement: Figure S3 — Correct genomic sequence of GmGγ5 as experimentally verified. (DOC) [file pone.0023361.s005.doc]

**Figure S3.**

**GmGγ5 (Gm11g18050.1)**

**ATGATAGCCATGGATGGACACCAACCACAACCATCATCAGAAACATTAGCATCAGAAAATGGGTGAGAATCAGAGGACTATGGAGAAGAAGGGGGAGAAAAGGGTCCTCATCCTCTTGCTCAAGCTGGAACTGGTTCTTTCCCTGGAGGCTTCATTGGGAAACATAGGTTGCAAGCAGCCATAACCAACCTCAATAACCAAATTAGTATTTTGCAGG**TCATCTCAACTCATCATGCCTCTTTTTTGTGTCAATTTGGTGATCCATCTTATTTTGTTCAATTTATATGCACAATTTTTTTCTCTTCATCTTAATTCTCCTAGAAGAAGAATTTGATAAATGAACTATGATGAGCAGG**AAGAATTGGAAAAAGTTGAAACAATTGGTGAATCCTCCACCGTTTGCAAGGA**GTAAGCAAAGCCAACATATAAGACTATGATATTATTATTCTTTCTTATGGTTCTTCAAACCTTAGAGTTTAATTATTGTTCATAATCTAAATTGAATTGCTCCATCCATTTTGTGCTCTTGCAG**TTTAATTTCAAGCGTTGAATCCATTCCAGATCCTCTCCTTCCATT**GTAAGAAATTAATCCTCTTGATGTTCATGATATTAATTTTAAGGGTCCAATGTTGCGTTGTATGTGTTTTTTAATGTACTAGTAAAGAAAGTAAATAATATATTATAATCCAAAAGGTAAAAAAAGAAAAGATATTTGAACAAAAATGATTAATGTATTAGAGTTAAGGTTAAGGTAAAATGTTTGTTTGCAG**CACCAAAGGTTCAGTTGATGCTGGTTGGGATCGATGGTTCGGAGGTGCCCACCACTCTCGAAATCACAAACGTTGGATTTAG**
