# Supplementary material for: Conventional and Novel Gγ Protein Families Constitute the Heterotrimeric G-Protein Signaling Network in Soybean
Source: PLoS One. 2011 Aug 10;6(8):e23361. doi: 10.1371/journal.pone.0023361 (PMC3154445; doi:10.1371/journal.pone.0023361)
Supplement: Figure S4 — Correct genomic sequence of GmGγ8 as experimentally verified. (DOC) [file pone.0023361.s006.doc]

**Figure S4.**

**GmGγ8 (Gm15g19630.1)**

**ATGAGCACTCCCACAAGAGGCACTTCTAATACGCTTCTCTCTCTGCCATTGCCCTCTCCTACTCCTAAGGCGCCTTCCGGGTACCCAGATTTGTATCGAAAGCGCCGCGAAACGGCCAAGATTCAGATGCTGGAAAGAGAGATAAGCTTCCTTGAGG**TTGGTTTCACTTTGTTAAACTGTAAATTCACCACTTAAACTCATACTCAAACTCAAAGGAAAAAATAAAGGGAGTCTAACTAATTAGATGAACAGTTACATGAGTTGTTTTAAGTTCTGTAATACCAATCTTTAATTCCTATAAATAAATAAAAACTCAAAGGAAAAGAAGTAAATAAAAGCACTACTTTGAACTTAGTTGAAATTCTCTTCACTTTGATTTTAAGAAATGGCATAATTACTTAGTACCTGTTACCATTACATTTCATGATCAAAACCAAACTTTAAAGTATGAAACAGAGTGTGAATGCACTGTTCATTGGCGATATGCGATTCATGAGTAGATAGACTTTCAACAGATAATTGCATGAATATAGGGTGTTCTTTAATTTTGCCAGTAGATTTTGCAATTGATTACCACTTGGAGTGATCTTATGCCTTTAGAGATTAATTTTAGATCAATGGAAAGAGTTAGAATTGAGTGAAAGATAATGTAATGGTTATAGATTGACTTTTTTCTGTGTTTCTTTACATTCGGTTTAATTTTTCTTTTTCATTTTATTTTAGGGGTATATGGGACCTAGCCTTTTTCACAACGGATATTAATTTTTTACACTACTGCATTATGCTTCTTTTCATATCCAATCATACTAAATTAGCATTTATTAATATTCAATTATTTACTGAATTATAAGTTGTAATTGTGAGCCAACCACTAGCATTTATTAAGCATGGAGAGGAAAAAAAGTAAAAACAGTGCCGAACTTTTAAACTGTGCTCCTTGGATGCCGATTGAGGGATTCACAGTAGACCATAGCTTCCTAGATTGTGAATCTTATGCAGTTATGGAATTGCATGCATGTTAAAATTATGTTAGCAATAATTTTTAAAATTATAAAATTAGCTTACCAAAATAAAAGTAATTTATAAAATTAAGAAAAAAGCAAAGAACGACACATTACATGCAAGCGTGATACCACTATCAATCTTGCAATTTTGCATTGAATTAATTTGTGATAGACACTTTAATTGAAGCCTTGTTAAATACCTTCCCTTTCTGGCCTCTTTGGTGTTAACCAAAATTTATTGACAATTTTGCATCAAACAGG**AAGAGTTAAAGTCTTCTGAAGGCCTTCAACCTGCTTCAAGATGCTGCAAAGAG**TATGTCTATTTGTGGAATTTCTTGTATAATATTATAATTATTGCTAGCTAAATTAACTTTGTTAATTGAATGCAACACTATTGCAGG**ATTGCCGATTTTGTGATGGCAAACTCAGATCCTCTGTTACCTAC**GTAAGAAATGCTCTTGCCAAACTTCCCTTGTCAGTTTAAAAATAAAAGATCATGATTATTATATCTTTCTCATTCAAGCAG**GAGCAAGAAGAACCACCAGTCATGTAGCTTGTGGAAGTG**GTTGTGGTACCCTTTTTTGTTAATTAATTTTGTTAGTTGATTTATTTAGTTAAAGGATTTTACTAACGGGTGTCGTAAATACATTGGTTAAAAAATTAAAAGAGAAAATGTTTTATTGAAAAATATCTAAGATTACATATAGAGGAATGCATTTTTATGCATCTCAAAAAAAAAAAAAATTTACTTCTTTAATGAGTGTCCTAATATCACCGGTGAGTAGTAACTATTTGGGATTCTGTGTAAAAAGCTATGTTAGAGTCTGATCTAGCTTTGATGGGTGACACGGACTTATGTCTAACTTTAAAAGATCATGCCTAGTTTTCTATCCCCTTTATCCTTAGTTTAGCTTCATACTAGCATACTGCTGAATCTGACAGTGTTGAATTCAACAACAACAAAAAAAAAAAAGAATCTAACATTGTTGTGTGTGTATCTGAGATGAACCAGATAATAATTCATAAGCTGCTTTATTTTCATTTCATAAACCAGCAACTTCTAACATAATAAACCTTGTACAATAGCTAATGATGCATGAATGCTTCAAAATTGTTGACAATCCCATACCACACCTGCACCATATACCTGAAGAACTTAAAGAAAAAACAATTTAAAAAATAAAATAAAAAATAGACTAAGGTTTTAAAATTATGAGTAACCATATCACTACTTTATTGAATAACATGAGAGTGGCCTTTTTGTGCAG**TGACATCCCTTGCTTTAACTTGTCTTGGATCTGCCATTGGTGCTGTGATGGGTGCTTTGAACATCAAAACTTGTCAAGTTGCTGTTCCCACGGCAAACCATGCAACTGCTGTTCTAGTTGTCTTCCATCTACCAATTGCTCTTGCTGCTCTAATGGAAGATCACATTGCTGCAAAGATAGCTGTGGTTGCAAAAATTGTTGCACTCTCCCAAGTTGCAATTTTGGGTGGCCTTTTCCCTCTTGCTGCATCTGCAAATGCTCTTGCTCTTGCTCTTGCCCAAAATGTCCCAAGGTTCGTCCATGTTGTTGTTGTACAAATTGCTGTTGGAACCCTTGTTCATGTTTCTAG**
