# Supplementary material for: Conventional and Novel Gγ Protein Families Constitute the Heterotrimeric G-Protein Signaling Network in Soybean
Source: PLoS One. 2011 Aug 10;6(8):e23361. doi: 10.1371/journal.pone.0023361 (PMC3154445; doi:10.1371/journal.pone.0023361)
Supplement: Figure S5 — Correct genomic sequence of GmGγ10 as experimentally verified. (DOC) [file pone.0023361.s007.doc]

**Figure S5.**

**GmGγ10 (Gm07g04510.1)**

**ATGGATGGTGGTGGCTACAACTTAACAACAACCTCTTTGAGCAGTTCAATGGAGCAAGTGATGAGACCCAAATCACCATTGCCGGGCTTAGTTGATTTTCATGGGAAGCGGAAGCAAATGGTTAAGATTCAGGTTTTGGAGAGAGAGATTGGTTTGCTTCAGG**TACATCTAAGAACACTTTTTTTTAAACTTTTTATCGCTTTGTGTAAATTCCTGTCTCCAAAATGTTCCTTCTTATTCTTTTGTTTTTCTCTTTTGCTATCTTCTCCAAACTGTTTACTTTTGTGTAAGATAAAAAGACTCGGAGTCTCAGACTGTTGTTATGTGAATTTTTTTTCCCAAACATACATAAAAATTAATTAAATACGAAAACATACATATCCTCAATTATTTACCGATATATATATATATATATATATATATATATATATATATATGTTTATTATTTACTGTATAAATTCAGTTTCGAGAACACGAATTTACTGTGTTTTTTCAAAAAAAATTTCAAAATCCCGTTTGAGAATTCGGTTCCTCCAATTCTCAACGTAATTTTTTTTTAATTTTAAGAAAAAATAATTTTTTAAGATTTAAAAATAATATAAATAAAAAAATTAATGTAAAATAATAAAATTAGAGAATATATTAAAGAGAAAAATAAAGTTAAAAAAATAGTTAAAAACACTTTCATTGAAGAATTTTTTTAAAATTATATATAATGTAAAATTAAATACATTGATAATAAATAAAAGAAAATTAATTTGCTTGATTAAAACTTTTACAACAATACATATTTTTTAATGACTTCTTACCCTGCAGATTTTGATTGACTTCTTTTTTTCTTATTCATATTAGTTTGTCTTTTTATTGATTTTGATTGATTTTTTTTTATTTCTCATAACTGAATTAACATATAATTGTGATCTTTTATATTAACACATAATTGTAGTCTTTCATATAGTGACTAACAATCATAATAAAATCCTAACACATAATTGTAGTCTTTCATATAATGACTAACAATCATAATAAAATCCTAACCGGTCTTTGTAGAACTCTACATATATGTCTCTGTCTTTCGTTGATGTGGTAACTATTTATTCAATCTGTTTTTAATCAATTTAATTTTTTAAATCATTTTCTTTTTAATTTATTTTAAGACTATTAATTAATTTTCTTATTTTTTTACTTAATATACCTAAGGAAAAAAAATCACCAACAAAACAAAAATTAAACTTTAGAAAAATTCATAATATGTTTAAATGTCTTAAGTTTCTAATTTATTTAAGTACATGATGATTAAAAAATTATATCATAATATTTTTTAATTTTATTAATTTTCTCTATTTATATTATTTTTAAATCTTTTAAAAAATTAATTTTTCTTAAAAAATTGACGTTCAGATTTTGGGTTGAAGGGATTCAAATTCTCAATCGGTTTTTGAATTTTTTTTTTGAAAAAACACTGTAAATTCGGGTTCTTTTAATGACACTGAATAATAAATGTGTATGTTTTGATCAATAATGAAAAATATGTATGTTTTGGTATTTAATTAATTTTTTATGTATATTCGGGGGAAAAAGATCCTTGTTATGTAGTTTGAGAAAGAATGAGCTACACTGTTTTTTAAAGCATCGATCATGCATTATGAAAGCTCTGTTTTAATAACTACATAATCACATTCCACGTGTTTTTGATACTGAAATGAGAAGTTTTTTTTGGATGGAATCTGTTTAATTAATTTGGTGGATGCAGAGGATTTCTATTGTTCGTATTAAATTTAGTTTTTAAACTCCCGAGTCTACCTATTGTTTTCATATAAAAAAAAAGTCTACGTATTGTACGATGTCGCTTTTAATTTAATGCGTTACAATAAACCAAATTATTTAACCATTTTGTTATGTGAAATTTATGCTAATCATGTGCTTCGGATTTGAAATATGTTAAGTTATTACTACAATAAAGGCGAGGCTTCATTGTCACATGGCTCTTCGTGATTTAGAGAAATTGAAACCATAGTTGTTTTCCTTGCTAAATTTTTAAGCAACCGTTATAGGCGCATTGTTTTAGTTAACTAGAAAATTGTTTTTAAACATAAGCTGGTGACTTATTTGTATGAAGGAGTAACGTTTAATTTCACTACAGAGTGATAGTGATACTCATGAGATGGTAACTGTATAATCCTCATCAGTCATCACATAATGTGTGGCACTAGTGTTTTGATTTCTTAATGGAAATTTGAGCTTCTGTTTGTGTTTATAACAAGTTGGTTTAAATATGTTTTCAATGTTATAAATATCACCTGTGTCATGTATATTGTTCCTAGG**AGGAATTAAAATCACTTGAAGGCCTTAACCCTGCTTCTAGATGCTGCAAAGAG**TAAGGCTCCCATCTTCACTATTGTCTACATTATTATATCCGATTCCAAGGTTTATTGAAAAATAAAATACAAATCATATTTGTATTAAAGTATTTTATATTCTAATTTGAAGTTAACGTTTTCCTTAACAGG**CTTGATGCCTTTGTAGACTCTGTTTCAGATCCCTTCACACCCAC**GTATGAGTCTTCAGCTTTCTCCATTTAATTTCTAGATTATTATTTTTTGTTACAAATCTCAGATAAATAATAAATCATTGTATCTGATACAG**AAGAAAACAGACAGTCTCTAAATCCCATCACTTCAGGAGGCAGATGATCAG**GTACACTACTTTGTCTTTTTACCCTTTTTTTCATTTTGATATGTTCTTTCACTTCCTTCCATTTCTAAAATTCATGATGCATAATTCAATGGAGTCATTTCTAAACAACAGTATTAGTACTTCCTCTCTTTTAACAGCTGAAGGAAACCGAAACATGAAGGGGCATGTGTTTTAAAAAACCTGTATTTTAATATATTTTGTATCATTAATAGAAATTTATACGAGACTCAATTCTCAAGAATAGAATAAAAAAATTAATCTCTTAATATTTTTTGTTTGTTTTAACGAATTAAAACATGATTATTTTTGTGTGCTATTAAAACATAATTCTCCTCTTTTTTATTTTTCTACATTTGACATCTCCCTACAATCTTTATCTTTGTCTTTTATTTACATATTATTTTATTAACAATTTTAACAAATTTAAATTTATTTAATATTATTTTTTATAATACCTAACTAACTCTCCGAACTTTATACATATGCCTTGGATATATTGCTGCAAACATACCTAATAATTATCATGTCTAATTAAAGAAAGACATAGCAATGGAACATGAGCAGTTTTTTTTTTTCTTTTCTAATACGACAGCAATATAAATAATTTAAATTATTTATATAATTTAGGTTTGAAATTGTCAGTTCCTATCATGATATTATAACTTTCATGAAGAAATAGTCTGAAATTTGATAATTCCTTTGTTGGTCTCTCTTAATAAAAAGTTGAGTTATAATACAAAATATTTTTAAAAAATAAAAATACATAAAAGTATATTTTTAAAATTTCTCGTTTGTAAGTAAGTTGACTATATGTTTTATTTTATTTACATAAACAAAAGAATAATAAGAAAATTTGAATGTTTCAATTTATAAAATTTTCATAAAATCAAATCTTATCAAATGAAACTAATTATACGATATCCTTAAGGAACTCAAATCCAAATTAACAAATGATGAACATTTTATTTAAAATTTACTAGTTAATTATCTAATAGTTGTGTCTAAATTAAAATGAGATGTTAGGTTTGTGCCTACTTAAAAATATCTCTATGTTCATAGGTTGTTATTCCAAAATCAAGTTGATGGTAAAATTTAGTATAAATTTTGAACCATCACAAAATCTATTTAAAAAGACACAATGTATTAAAAGGTCTAATTCAATTAATTAAATGAAACAATGTGAATATGGGCAACTTATTAAAAGCTATCTATTATCTGAGTTCACTTTATATGAATAAATAAAAAGAAGACACAATGTTGTGCAAGTATCGACACGACAAATGAATAACGAGGACGTTAACTTTCTTTTATTTTTCTCAG**TCTTCCACGGGTTTGCTGCTCCAACAGTTGCCTGCTGCATAAGAAAACAGCAAAAGGTTGTTGTTGGGACTGCTGTTCTTCATCAAACTCCAAGTGTTGTCACTGTTGTGGCTGCTGCTGCTGCTGCTTGAAGAGTGCATCAAGATCCTGTGCTCAAAATTGTTGCTAG**
