# Supplementary material for: Conventional and Novel Gγ Protein Families Constitute the Heterotrimeric G-Protein Signaling Network in Soybean
Source: PLoS One. 2011 Aug 10;6(8):e23361. doi: 10.1371/journal.pone.0023361 (PMC3154445; doi:10.1371/journal.pone.0023361)
Supplement: Figure S6 — Evolutionary relationships of GmGγ proteins. (PPT) [file pone.0023361.s008.ppt]

## Slide 1
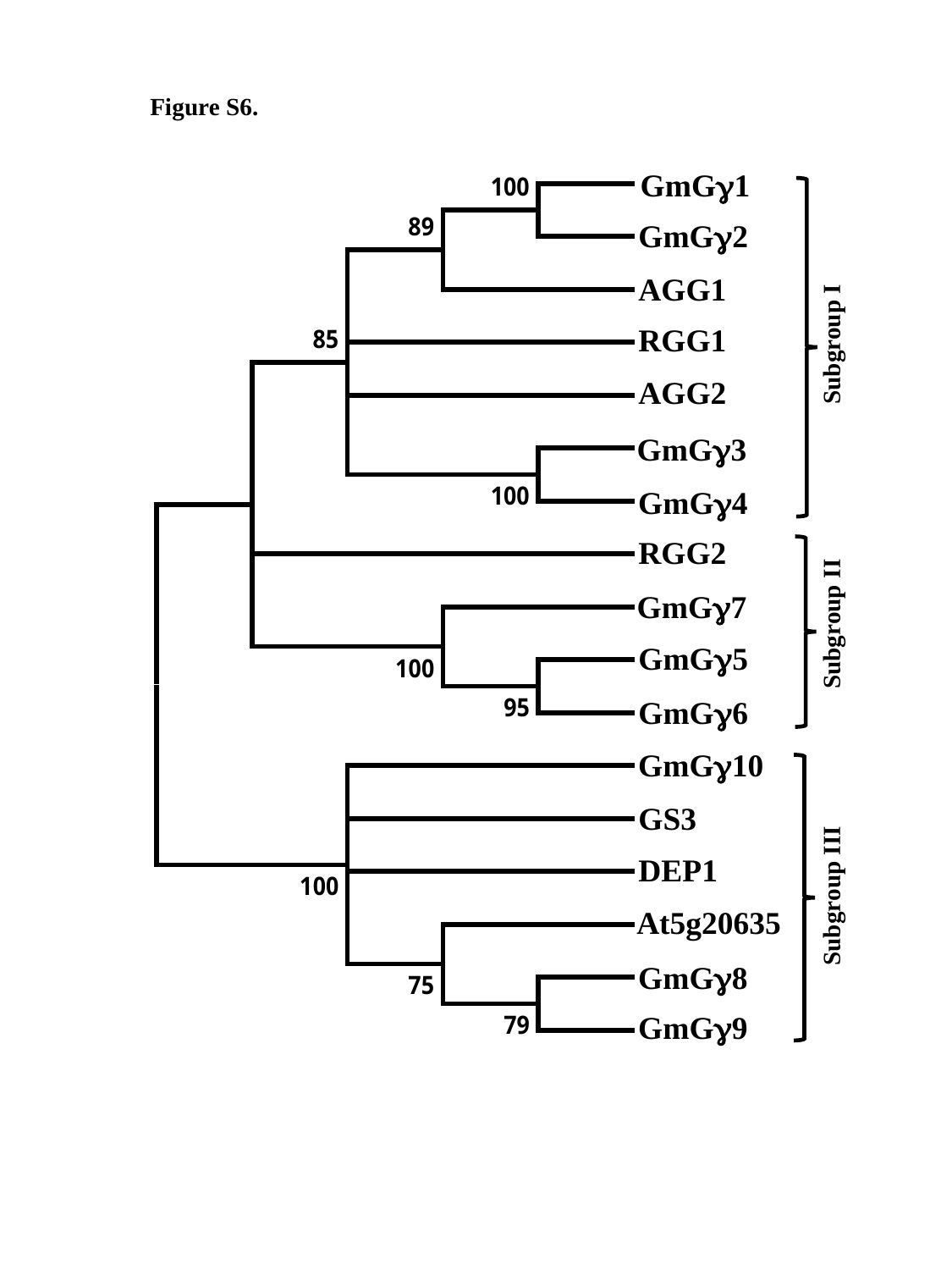

Figure S6.
GmGγ1
GmGγ2
AGG1
RGG1
Subgroup I
AGG2
GmGγ3
GmGγ4
RGG2
GmGγ7
Subgroup II
GmGγ5
GmGγ6
GmGγ10
GS3
DEP1
Subgroup III
At5g20635
GmGγ8
GmGγ9
