# Supplementary figures and images for: Conventional and Novel Gγ Protein Families Constitute the Heterotrimeric G-Protein Signaling Network in Soybean
Source: PLoS One. 2011 Aug 10;6(8):e23361. doi: 10.1371/journal.pone.0023361 (PMC3154445; doi:10.1371/journal.pone.0023361)

## Slide 1
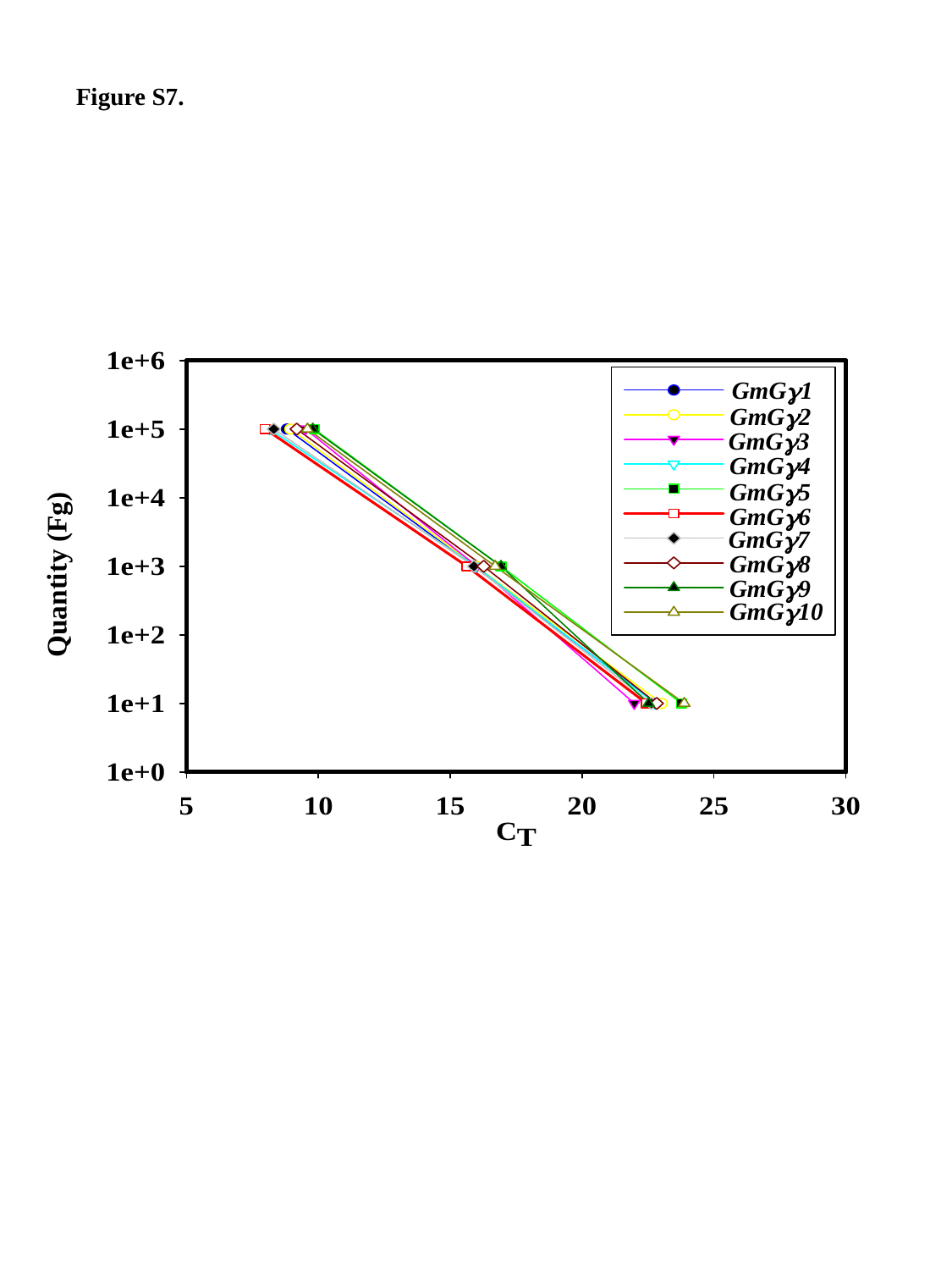

Figure S7.
GmGγ1
GmGγ2
GmGγ3
GmGγ4
GmGγ5
GmGγ6
GmGγ7
GmGγ8
Quantity (Fg)
GmGγ9
GmGγ10

Supplement: Figure S7 — PCR efficiency of GmGγ genes over 100,000 fold dilution. (PPT) [file pone.0023361.s009.ppt]
