# Supplementary material for: Conventional and Novel Gγ Protein Families Constitute the Heterotrimeric G-Protein Signaling Network in Soybean
Source: PLoS One. 2011 Aug 10;6(8):e23361. doi: 10.1371/journal.pone.0023361 (PMC3154445; doi:10.1371/journal.pone.0023361)
Supplement: Figure S8 — Test of interaction between the N-terminal and C-terminal parts of GmGγ 8, 9 and 10 proteins with different GmGβ proteins. (PPT) [file pone.0023361.s010.ppt]

## Slide 1
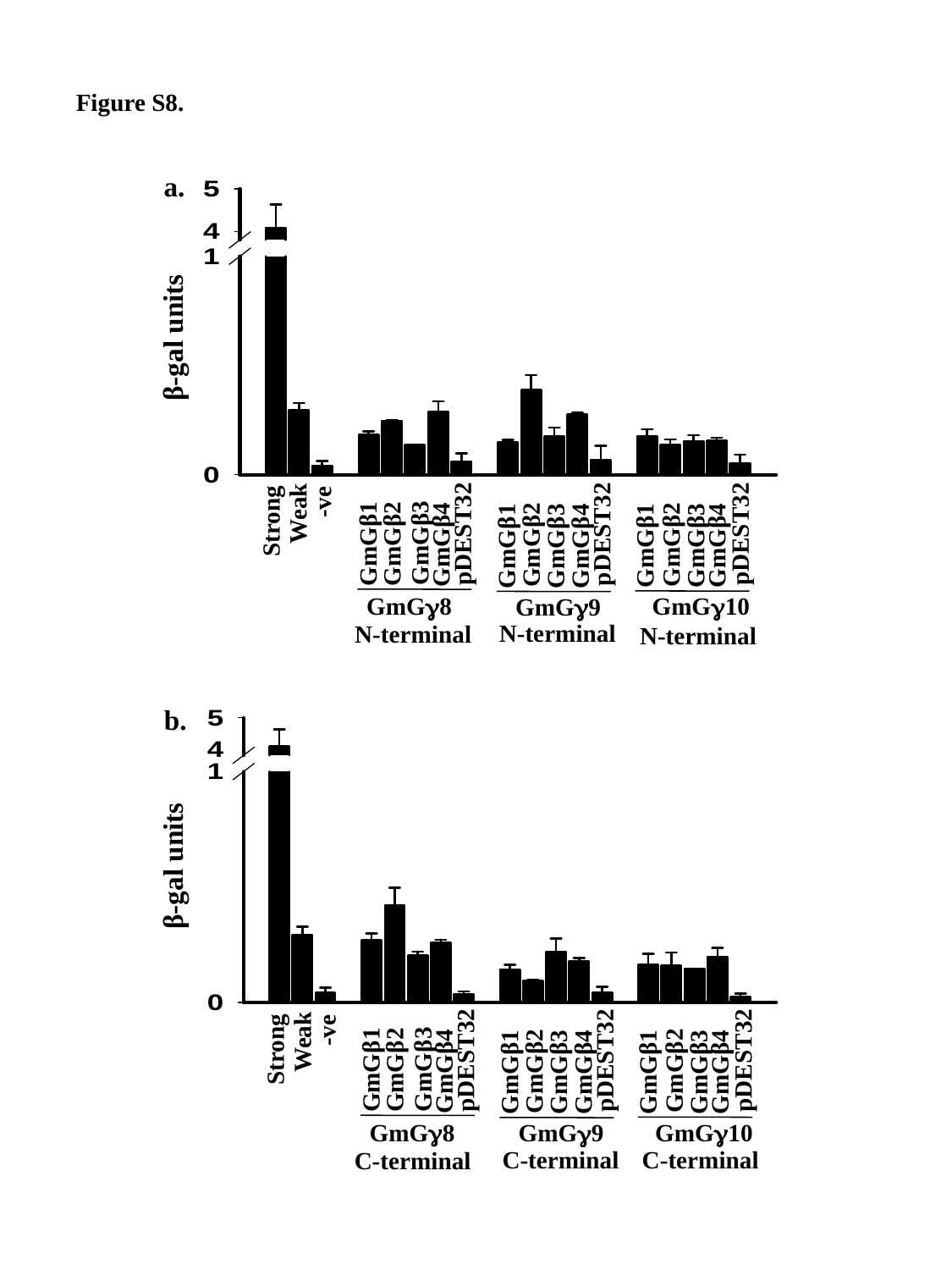

Figure S8.
-ve
Weak
GmGβ3
GmGβ1
GmGβ2
GmGβ2
GmGβ4
GmGβ2
GmGβ1
GmGβ3
GmGβ4
GmGβ1
GmGβ3
GmGβ4
Strong
pDEST32
pDEST32
pDEST32
GmGγ8
GmGγ10
GmGγ9
N-terminal
a.
β-gal units
N-terminal
N-terminal
-ve
Weak
GmGβ3
GmGβ1
GmGβ2
GmGβ2
GmGβ4
GmGβ2
GmGβ1
GmGβ3
GmGβ4
GmGβ1
GmGβ3
GmGβ4
pDEST32
Strong
pDEST32
pDEST32
GmGγ8
GmGγ10
GmGγ9
C-terminal
b.
β-gal units
C-terminal
C-terminal
